# A rapidly-reversible absorptive and emissive vapochromic Pt(II) pincer-based chemical sensor

**DOI:** 10.1038/s41467-017-01941-2

**Published:** 2017-11-27

**Authors:** M. J. Bryant, J. M. Skelton, L. E. Hatcher, C. Stubbs, E. Madrid, A. R. Pallipurath, L. H. Thomas, C. H. Woodall, J. Christensen, S. Fuertes, T. P. Robinson, C. M. Beavers, S. J. Teat, M. R. Warren, F. Pradaux-Caggiano, A. Walsh, F. Marken, D. R. Carbery, S. C. Parker, N. B. McKeown, R. Malpass-Evans, M. Carta, P. R. Raithby

**Affiliations:** 1 Department of Chemistry, University of Bath, Claverton Down, Bath, BA2 7AY UK; 2 The Cambridge Crystallographic Data Centre, 12 Union Rd, Cambridge, CB2 1EZ UK; 3 Research Complex at Harwell, Rutherford Appleton Laboratory, Harwell Oxford, Didcot, Oxfordshire OX11 0FA UK; 4 Advanced Light Source, Lawrence Berkeley National Laboratory, 1 Cyclotron Road, Berkeley, CA 94720 USA; 5 Beamline I19, Diamond Light Source, Harwell Science and Innovation Campus, Didcot, Oxfordshire OX11 0QX UK; 6 Global E3 Institute and Department of Materials Science and Engineering, Yonsei University, Seoul, 120-749 Korea; 7 EastChem, School of Chemistry, University of Edinburgh, Edinburgh, EH9 3FJ UK

## Abstract

Selective, robust and cost-effective chemical sensors for detecting small volatile-organic compounds (VOCs) have widespread applications in industry, healthcare and environmental monitoring. Here we design a Pt(II) pincer-type material with selective absorptive and emissive responses to methanol and water. The yellow anhydrous form converts reversibly on a subsecond timescale to a red hydrate in the presence of parts-per-thousand levels of atmospheric water vapour. Exposure to methanol induces a similarly-rapid and reversible colour change to a blue methanol solvate. Stable smart coatings on glass demonstrate robust switching over 10^4^ cycles, and flexible microporous polymer membranes incorporating microcrystals of the complex show identical vapochromic behaviour. The rapid vapochromic response can be rationalised from the crystal structure, and in combination with quantum-chemical modelling, we provide a complete microscopic picture of the switching mechanism. We discuss how this multiscale design approach can be used to obtain new compounds with tailored VOC selectivity and spectral responses.

## Abstract (web summary)

Solid state Pt(II)-pincer complexes exhibiting vapochromic responses show promise for chemical sensing applications, but their slow responses typically limit their utility. Here, Raithby and colleagues design a Pt(II)-pincer complex with a subsecond, highly-selective vapochromic response to water and methanol.

## Introduction

Small-molecule chemical sensors have important technological applications ranging from industrial process monitoring, to medical diagnostics (e.g., the identification of disease biomarkers in exhaled breath^1^), home security (e.g., explosives detection) and environmental surveying^2^. An ideal sensing platform should show a robust, highly selective and unambiguous response. It should also be cheaply and easily deployed in a simple device architecture, without requiring a complex readout system.

Phosphorescent square-planar platinum complexes are an active area of research in this field^3^, due to numerous systems showing solid-state vapochromic responses to small-molecule analytes^4–7^. The solid-state photophysical properties of these materials arise from intermolecular metal-metal interactions. Minor changes to the local environment of the molecules in the solid state, e.g., due to external stimuli such as temperature^8^, pressure^9, 10^, exposure to chemical vapour^3, 4, 11, 12^, or mechanical stress^13^, can perturb these interactions and induce substantial changes in absorption and emission properties.

Square-planar cyclometallated Pt(II) polypyridine pincer complexes are of particular interest as their luminescence, and the ease with which it can be manipulated, are well established^14–21^. The polypyridine ligand incorporates significant scope for modification, with numerous potential and synthetically-accessible substitution sites. By varying the ligand chemistry, compounds with emissive frequencies across the visible spectrum have been reported^22–24^. The monodentate ancillary ligand L, occupying the fourth Pt coordination site, likewise plays an important role in the solid-state aggregation and intermolecular interactions^25, 26^.

The gas-sensing properties of Pt(II) pincer complexes in the solid state were first exploited by van Koten et al.^27^ in their pioneering work on the uptake of SO_2_ by [PtCl{C_6_H_2_(CH_2_NMe_2_)_2_−2,6-OH-4}]. When exposed to SO_2_ gas, the initially colourless crystalline powder was transformed to a deep orange colour over a period of minutes, an effect only previously observed in solution. SO_2_ could subsequently be removed by a flow of N_2_ gas, confirming it to be a reversible process.

Other notable examples of solid state Pt(II)-pincer complexes exhibiting vapochromic responses include [Pt(Me_2_bzimpy)Cl]PF_6_ (Me_2_bzimpy = 2,6-bis(*N* ^−^methylbenzimidazol-2-yl)pyridine), which crystallises as an orange dimethylformamide (DMF) solvate that turns purple on the order of seconds when exposed to acetonitrile vapour^4, 5^. Similarly, the related [Pt(Me_2_bzimpy)Cl]Cl responds to methanol, ethanol, chloroform and acetonitrile, displaying a colour change from orange to red^4^. [Pt(Nttpy)Cl] (Nttpy = 4′-(p-nicotinamide-N-methylphenyl)-2,2′:6’,2′′-terpyridine) is a brightly-luminescent solid at room temperature that changes from red to orange on uptake of methanol^28^. In all three cases, the colour switching is attributed to changes in the stacking arrangement within the crystal, leading to a change in the Pt…Pt interaction. In addition to pincer systems, Pt(II)-complexes with bidentate aromatic ligands also show vapochromic responses on exposure to water and various organic solvents^6, 7^.

Crystallographic studies have established that crystals of pincer complexes typically possess channels that allow the diffusion of small molecules through the lattice, disturbing the Pt…Pt stacking (and $d_{z^2}$ overlap) and inducing an optical response^29, 30^. This provides a means to control the chemical selectivity through the ligand chemistry and the crystal packing. The ancillary ligand L plays an important role here, as functional groups on this ligand project into the channels and can interact with guest molecules.

The proposed mechanism for the optical response is that the uptake of guest molecules induces changes in the Pt…Pt interactions and hence perturbs the electronic structure^4^. However, a direct link between changes in the crystal and electronic structures has not yet been established, which is essential to further the rational design of materials with tailored chemical selectivity and optical responses. Furthermore, most studies have focussed on proof-of-concept measurements demonstrating vapochromic behaviour, with little investigation of long-term switching durability or prototype device architectures. Both technical questions must be addressed as a prerequisite to eventual device applications.

We have designed a vapochromic Pt(II)-pincer complex that exhibits a selective, subsecond absorptive and emissive response to water and methanol. The response spans the visible spectrum, switching reversibly from a yellow desolvated form to either of a red hydrated or a blue methanolic form. The compound is easily deployed as a stable smart coating on glass, which shows a reversible response over > 10^4^ switching cycles. It can also be impregnated into a flexible polymer membrane that then shows the same behaviour. Complete characterisation *via* a combination of spectroscopy, X-ray crystallography and quantum-chemical modelling has allowed us to relate the macroscopic colour changes to structural perturbations at the molecular level, unambiguously elucidating the microscopic switching mechanism. Analysis of the crystal structure also explains the unusually rapid vapochromic response, which is unprecedented in this type of system. Finally, we discuss how a combination of ligand design and materials engineering can be used to develop new materials for specific sensing applications.

## Results

### Design and synthesis

**Fig. 1:**
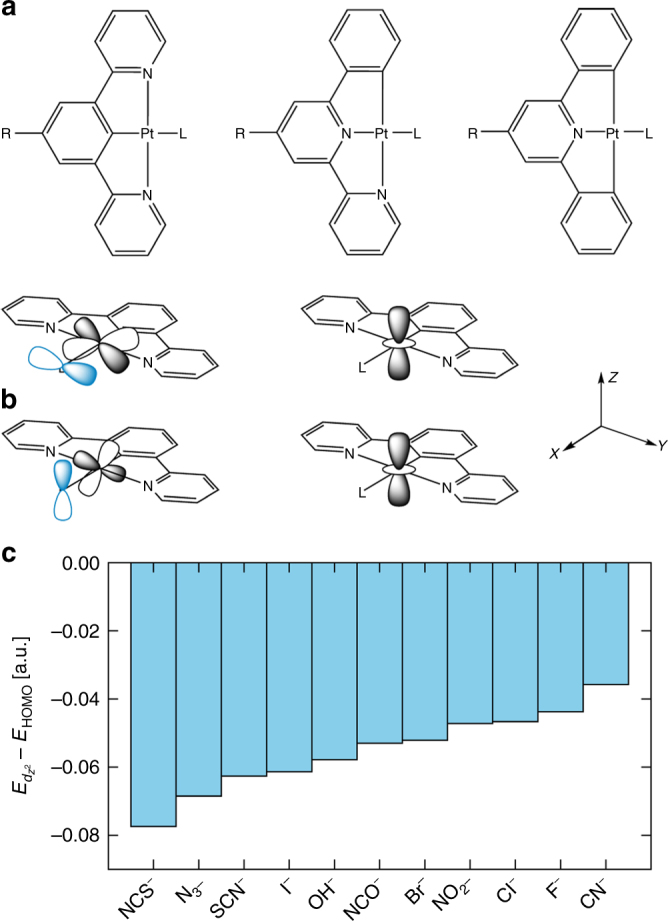
Structure and electronic properties of Pt-pincer complexes. **a** Structure of general Pt-polypyridine pincer complexes with N^C^N (left), N^N^C (centre) and C^N^C (right) pincer ligands. **b** The valence Pt $d_{xy}$ and $d_{xz}$ orbitals interact strongly with *π*-type molecular orbitals on the monodentate ancillary ligand (left), while the $d_{z^2}$ orbitals of adjacent molecules can interact in the stacked geometry typically adopted in the solid state (right). **c** By adjusting the ancillary ligand, the energy of the $d_{z^2}$ orbital relative to the highest-occupied molecular orbital (HOMO) can be manipulated, making the optical properties more sensitive to the solid-state stacking

The starting point for our design strategy is the planar skeleton 1,3-di(2-pyridyl)benzene tridentate (N^C^N) Pt(II)-pincer complex (Fig. 1a, left), which has shown high quantum yields and greater promise for luminescent applications than similar N^N^C and C^N^C complexes (Fig. 1a, centre/right)^14, 22, 31^.

The valence metal $d$ orbitals in these square-planar Pt(II) complexes are high lying^32^. The $d_{xy}$ and $d_{xz}$ orbitals have the correct symmetry to interact with *π*-type orbitals on the monodentate ligand, while the $d_{z^2}$ orbitals on adjacent molecules can interact in the typical molecular stacks adopted by these complexes in the solid state (Fig. 1b)^33, 34^. The $d_{z^2}$ overlap produces high-energy crystal orbitals and makes the electronic structure sensitive to the crystal packing. Quantum-chemical calculations (see Methods section and Supplementary Note 1) show that by changing the monodentate ligand L the energy of the $d_{z^2}$ orbital can be raised towards the frontier in the molecular complex (Fig. 1c). Varying L for a given polypyridine therefore provides a means of fine-tuning the primary absorption and the vapochromic response due to changes in the Pt…Pt stacking. Subtle differences in the Pt…Pt stacking in response to structural perturbations effectively adjusts the energy of the crystal orbitals formed by the $d_{z^2}$ overlap, and hence the range of the energy gap between the highest-occupied and lowest-unoccupied crystal orbitals (HOCO/LUCO).

The (N^C^N) ligand type has an advantage over the related teryridine ligands (N^N^N) in that with a monoanionic ligand, L, in the fourth coordination site, the complex is neutral, thereby circumventing the need for a counterion that may disrupt the stacking within the crystalline state. We selected a methylester as the R substituent, firstly to improve solubility and crystallisation, and secondly for its potential to form additional intermolecular interactions, which have previously proven beneficial in promoting the formation of solvent-accessible channel structures in the solid state^28^. Cyanide was selected as the ancillary ligand L with the following rationale. Firstly, the quantum-chemical calculations in Fig. 1c indicate that, of a range of potential synthetic targets, CN is expected to bring the $d_{z^2}$ orbitals towards the frontier and thus to raise the energy of the crystal HOCO; this in turn should shift the primary absorption and vapochromic response into the visible region. Secondly, the polarity of the CN ligand provides a means for polar guest molecules to interact with the host structure, disturbing the molecular stacking and inducing a spectral response.

**Fig. 2:**
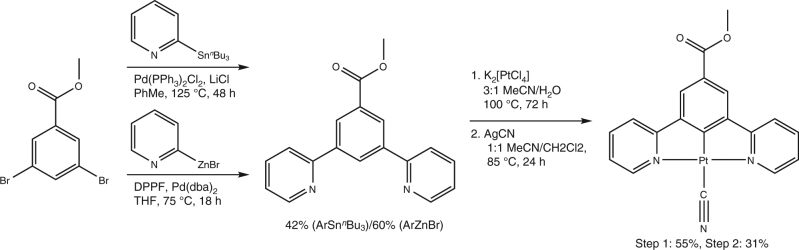
Synthesis of Pt-pincer complex **1**. Two alternative routes for preparing the pincer ligand methyl 3,5-di(2-pyridyl)benzoate were tested, based on Stille (top) and Negishi (bottom) coupling reactions

The synthesis of the target complex **1** is reported in Fig. 2 and detailed in the Methods section. The pincer ligand methyl 3,5-di(2-pyridyl)benzoate and corresponding chloro-Pt complex chloro[methyl 3,5-di(2-pyridyl)benzoato]platinum were initially prepared using an adapted procedure from the literature^35^.

The synthesis of the ligand can be performed using a Stille coupling, but an alternative Sn-free route under milder conditions, based on a Negishi coupling, was found to afford a higher yield^36^. The precursor chloride complex is obtained by reacting the pincer ligand with K_2_[PtCl_4_], and complex **1** is formed by further reaction with AgCN. The overall yield across the three steps was 7 and 10 % using the Stille or Negishi routes, respectively.

### Vapochromic response

**Fig. 3:**
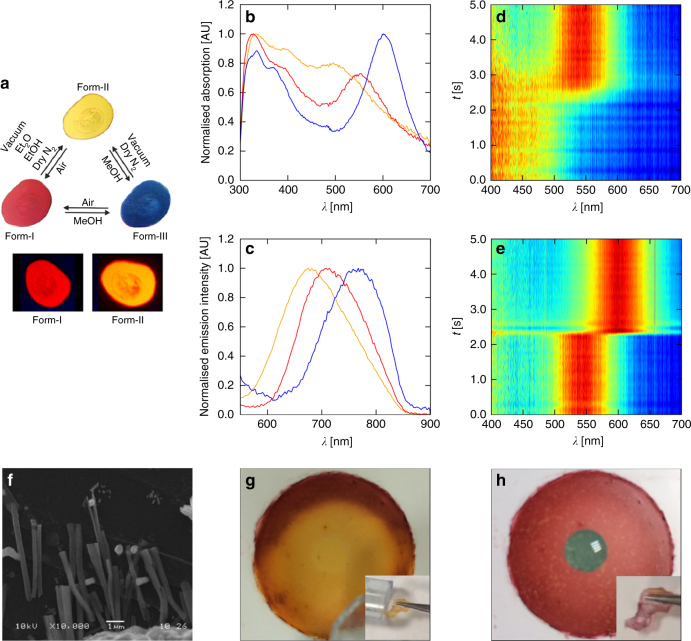
Solid-state vapochromic switching in Pt-pincer complex **1**. **a** Images of a thin film of **1** on glass in its hydrated (Form-I), anhydrous (Form-II) and methanolic (Form-III) forms, showing the reflective (top) and emissive (bottom) colours. **b**, **c** Solid-state absorption (**b**) and emission (**c**; 500 nm excitation) spectra of the three forms. **d**, **e** Time-resolved solid-state absorption spectra of **1** during switching between the anhydrous and hydrated (**d**; Form-II → Form-I) and the hydrated and methanolic (**e**; Form-I → Form-III) forms. The colour scale runs from blue (minimum) to red (maximum), and each spectrum used to build the 2D plot is individually normalised to emphasise the shifts in the absorption maxima. The artefact in e during the switching is the point at which the methanol drop was applied to the film. **f** Scanning-electron micrograph of a similar film to those in a prepared on carbon tape, showing the microstructure. **g**, **h** Vapochromic switching in flexible polymer membranes impregnated with **1** on a support (main) and free standing (inset)

A 10^−6^ M solution of **1** in dichloromethane is pale yellow and displays strong luminescent emission at 477 nm when excited at 395 nm (Supplementary Figs. 1–3). This was assigned as originating from a ^3^LC state with reference to related complexes^29, 37–39^. Evaporation of the solvent in ambient air produces a vivid-red microcrystalline solid (Form-I; Fig. 3a). Under a flow of inert dry gas, a drop-cast film of Form-I *ca*. 2 cm in diameter displays a rapid change to a yellow anhydrous Form-II, reverting immediately to Form-I upon interruption of the flow. This instantaneous colour change results from the purging and reabsorption of atmospheric water vapour by the crystallites in the film, and the same response is induced by heating or by placing the film in vacuum.

Exposing the films to liquid methanol or methanol vapour induces a similarly-rapid transition to a third, deep-blue Form-III, which is again rapidly and fully-reversible either by drying in air (yielding red Form-I) or under dry nitrogen (initially yielding Form-I, from residual water in the methanol, followed by rapid conversion to Form-II). Videos of the colour changes can be found in Supplementary Movies 1–3. The Form-I films showed no sensitivity to a range of other solvents, including ethanol, diethyl ether and acetone, apart from gradually turning yellow in some of the dry, water-miscible solvents due to the slow diffusion of water out of the crystals.

Notably, replacing the CN^−^ group in **1** with other anions such as OH^−^ or NO_2_^−^ did not lead to similar, fast vapochromic behaviour. We attribute the unique properties observed with the CN^−^ ligand to the electronic properties described above, and also to the overall planarity of the complex and the hydrogen-bond acceptor properties of CN^−^, which facilitate the interaction with water and methanol.

Colour switching between the three forms was quantified by solid state UV-visible absorption spectroscopy (Fig. 3b). All three spectra display strong, broad absorption bands in the visible region, and the transitions between the three forms are accompanied by significant wavelength shifts of the lowest-energy absorptions, in accordance with the observed colour changes. The absorption maximum of Form-II is blue-shifted to ~500 nm with respect to that of Form-I (560 nm), whereas Form-III has a red-shifted maximum at ~600 nm.

As in solution, all three solid forms are also emissive (Fig. 3a, c), with the order of emission wavelengths mirroring that of the absorption maxima. The peak emissions of the anhydrous Form-II and hydrated Form-I occur at ~670 nm and 720 nm, respectively, producing orange and red emissive colours, while the emission of the methanolic Form-III peaks around 770 nm. This infrared emission is notable given the comparative rarity of organometallic IR emitters.

To study the kinetics of the vapochromic switching, we exposed an initially anhydrous film, suspended under a nitrogen stream, to water vapour (Form-II → Form-I**)** and then to methanol (Form-I → Form-III) while monitoring the changes in the absorption profile using time-resolved UV-visible spectroscopy (Fig. 3d, e). From measurements made at 100 ms time resolution, both transitions were found to proceed on subsecond timescales, which is notably faster than other reported vapochromic Pt(II)-pincer systems^4^. To the best of our knowledge, the only other system to exhibit such a rapid response is [Au(im(CH_2_Py)_2_)_2_(Cu(MeOH))_2_][PF_6_]_3_ (im = imidazole), an *N* ^-^heterocyclic carbene complex that switches to green luminescence under UV excitation on replacement of the methanol by acetonitrile^40^.

A similar preparation to the glass slides was carried out on carbon tape and studied by scanning-electron microscopy (SEM; Fig. 3f). This showed that the films consist of microcrystalline needles ~5 μm in length and 0.2–0.5 μm in diameter.

### Towards device applications

The clear visual response of **1** to water, and the easy deployment as a coating on glass, are ideal for application as a humidity sensor. Using a LiCl salt test and diffuse-reflectance spectroscopy (Supplementary Figs. 4–7), we identified the Form-II ↔ Form-I transition (i.e. yellow-to-red) to occur over a range of 4,500 to 6,000 p.p.m.V of water vapour. To quantify the durability of the switching, a coated glass slide was repeatedly passed through a dry nitrogen stream, inducing the Form-I ↔ Form-II transition, at a rate of 0.6 Hz (Supplementary Fig. 8 and Supplementary Movies 4 and 5). After continuous cycling over a period of 6 h ( > 10^4^ cycles), we observed no obvious degradation in the speed or colour intensity of the response. Similarly, drop-cast films stored under ambient conditions retained their vapochromic behaviour for >6 months, again with no significant degradation in the response.

We were also able to prepare flexible polymer membranes by drop-casting solutions of **1** with the PIM-EA-TB polymer of intrinsic microporosity (PIM)^41^ onto a polyethylene terephthalate (PET) support (Fig. 3g, h; see Methods section). The membranes showed the same vapochromic response as the films on glass, both when supported and free-standing (Fig. 3g, h, inset). Optical micrographs of supported films indicated that the membranes contain embedded microcrystallites similar to those observed in the glass coatings, but up to 10–20 μm in length (Supplementary Fig. 9).

### Structural origins of the fast optical switching

**Fig. 4:**
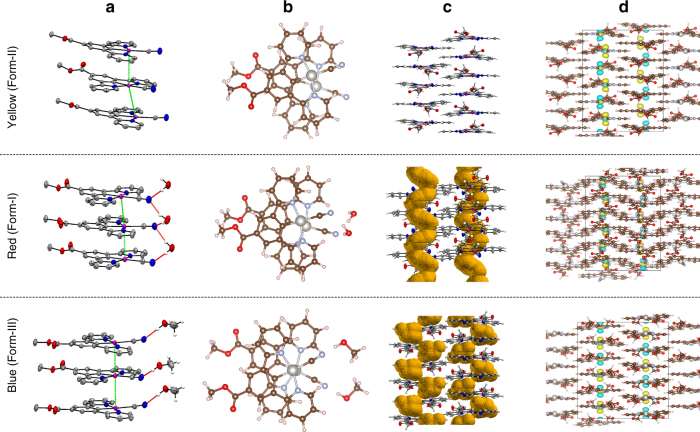
Solid-state stacking, channels, and orbital overlap in **1**. The yellow anhydrous Form-II ((R-N^C^N)Pt(CN)), the red hydrated Form-I ([(R-N^C^N)Pt(CN)].H_2_O) and the blue methanolic Form-III ([(R–N^C^N)Pt(CN)].MeOH) (R = COOMe) The single-crystal X-ray structures show how H-bonding interactions between the CN ligand and guest molecules decrease the Pt–Pt distance and lead to enhanced overlap between the metal centres (**a**, **b**). Analysis of the solvent voids (**c)** shows that the water molecules in Form-I form a continuous helical channel along the stacking direction, whereas the methanol molecules in Form-III adopt discrete pockets. Rotation of the Pt-pincer molecules in Form-II acts to fill the voids. Quantum-chemical calculations indicate the highest-occupied crystal orbitals (HOCOs) to be comprised of antiphase chains of Pt ${\it{d}}_{{\it{z}}^2}$ orbitals (**d)**, revealing the origin of the red shift in the absorption to be due to destabilisation of the HOCO arising from increased Pt–Pt overlap

The three crystalline forms of **1** were further studied by single-crystal X-ray diffraction using synchrotron radiation (Fig. 4, Supplementary Figs. 10–12 and Supplementary Table 1). A comparison of the atomic-scale structures of the three forms explains the structural origin of the optical switching and, crucially, provides a rational explanation for the unusually rapid vapochromic response.

Red single-crystals of the hydrated Form-I were grown from acetonitrile solution by slow evaporation in ambient air, while blue single-crystals of the methanolic Form-III were obtained by slow-cooling in methanol. In both cases, X-ray powder diffraction patterns simulated from the single-crystal X-ray data were consistent with those collected from the bulk powder samples (see Supplementary Figs. 13–15).

Various needle-like yellow crystal forms were obtained by crystallising **1** in a range of other solvents, of which only slow evaporation of a dilute acetone solution under dry nitrogen produced crystals with a structure consistent with the bulk powder sample of Form-II. Single-crystal-to-single-crystal transformations from Form-I to Form-III could be effected slowly by placing crystals of the hydrated form in a 5:1 mixture of acetonitrile and methanol for 2 weeks. Exposure to pure methanol resulted in rapid solvent uptake and fragmentation of the crystal. Similarly, conversion of Form-III to Form-I, which occurred rapidly when the Form-III crystals were exposed to ambient air, resulted in crystal fragmentation.

Attempts to obtain yellow Form-II by single-crystal-to-single-crystal transformation from the other forms likewise invariably resulted in loss of crystallinity due to the stress induced by rapid purging of the guest molecules. Given the stable switching observed over many cycles in the films, we infer that the stress induced by the transitions can be tolerated in smaller crystallites, which thus retain the solvent selectivity and optical response.

**Fig. 5:**
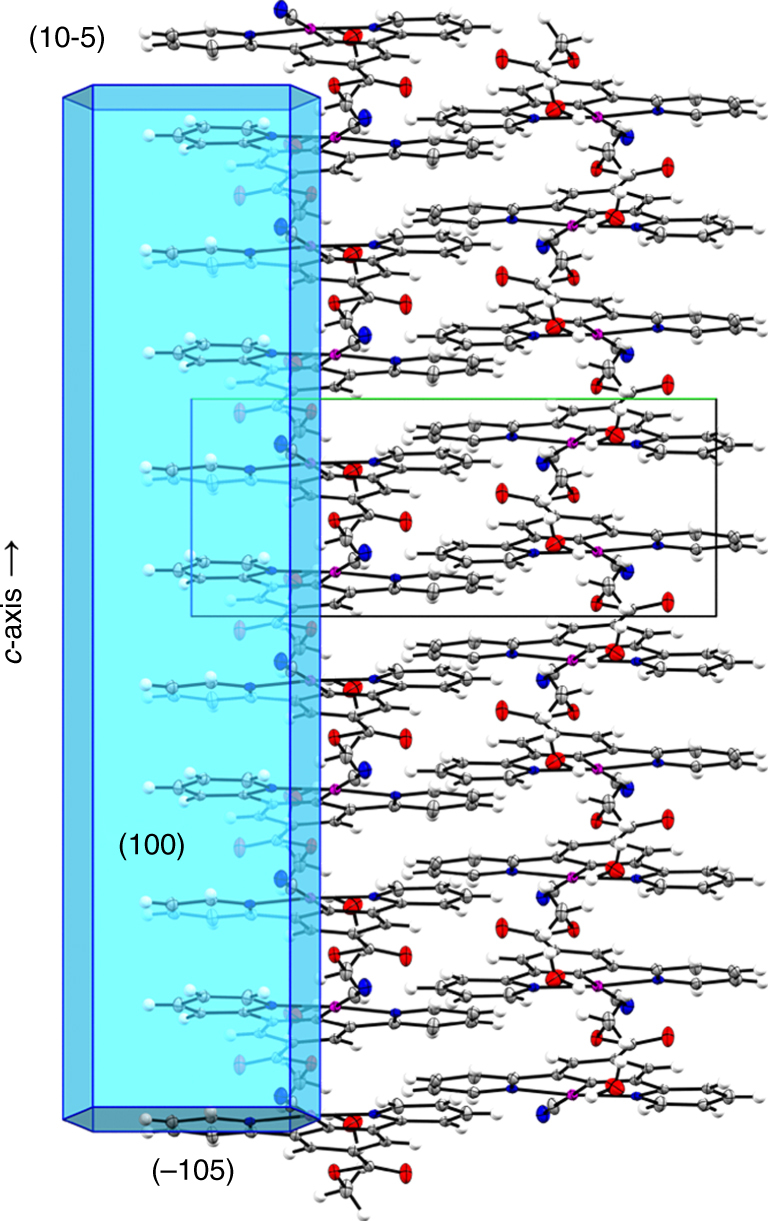
Solid-state Pt–Pt stacking in Form-I. The image shows the reconstruction of the natural crystal faces superimposed onto the crystal structure of Form-I, which confirms that the long axis of the needle-like crystallites is coincident with the Pt…Pt stacking direction (*c* direction, vertical). The crystal faces were determined by face-indexing a red needle of Form-I

The spectral properties of **1** are conveniently explained in terms of the Pt…Pt intermolecular interactions in each of the three crystal structures. All three forms crystallise in the monoclinic space group *P*2_1_/*n*, with 1D columnar stacks of planar Pt-complex molecules extending parallel to the crystallographic *c*-axis (Figs. 4 and 5).

The crystal structure of Form-I confirms the presence of solvated water molecules, which create an extended hydrogen-bonding network that bridges between the CN^−^ ligands on neighbouring Pt-complexes. This effectively ties adjacent pincer molecules into a relative orientation that brings the Pt-centres close enough to interact, forming continuous molecular wires. Stacked Pt atoms are separated by a Pt…Pt distance of 3.452(3) Å, indicating a degree of metal-metal interaction known to facilitate triplet metal-metal-to-ligand charge-transfer (^3^MMLCT) in related compounds^42, 43^. However, adjacent Pt-centres are not directly overlapped. Instead, neighbouring pincer molecules adopt a slight zig-zag arrangement (∠Pt–Pt–Pt 154.17(3) °), whereby the Pt-centres are staggered with respect to one another (∠(CN)–Pt–Pt–(CN) 33.52(11) °).

X-ray analysis of Form-II confirms the expected un-solvated structure, with no guest-mediated hydrogen-bonding network. This allows for a slight planar shift between alternate complex molecules, resulting in a more staggered arrangement of Pt-centres along the stacking direction and an increased Pt…Pt distance (∠Pt–Pt–Pt 142.48(2) °, Pt1–Pt1 3.663(1) Å).

The crystal structure of Form-III confirms the presence of methanol solvent molecules, which again participate in intermolecular hydrogen-bonding. In this case, each molecule forms only one discrete interaction with a nearby CN^−^ ligand, leading to further subtle changes in the Pt…Pt stacking. The metal-metal interactions benefit from the near-perfect overlap of the stacked molecules (Fig. 4b), facilitating the shortest Pt…Pt distance across all three forms of 3.388(1) Å and a near-linear chain of interacting Pt-centres along the stacking direction (∠Pt–Pt–Pt 178.41(1) °).

These subtle variations in both the Pt…Pt stacking distance and metal overlap would be expected to have a pronounced effect on ^3^MMLCT transitions in each of the three forms, providing a mechanism for the vapochromic response. This was qualified by first-principles electronic-structure calculations within the density-functional theory (DFT) formalism, which show that the HOCOs of all three forms are composed of antiphase chains of Pt $d_{z^2}$ orbitals (Fig. 4d). The effect of decreasing the Pt…Pt distance and aligning the orbitals is to enhance this antibonding interaction, destabilising the HOCO and decreasing the HOCO-LUCO energy gap. The LUCOs comprise extended in-phase interactions between delocalised pincer-based π* orbitals, and decreasing the intermolecular distance thus has the secondary effect of stabilizing the LUCO and further reducing the HOCO-LUCO gap (Supplementary Figs. 16 and 17). This provides an intuitive link between the structural changes and the observed colour shifts, and these conclusions were further verified by modelling the absorption spectra of the three structures (Supplementary Fig. 18).

Structural analysis of Form-I and Form-III also provides insight into the solvent-exchange mechanism, enabling rationalisation of the subsecond vapochromic response. The crystal structure of Form-I contains one solvent molecule per pincer complex, which was confirmed by thermogravimetric analysis (Supplementary Fig. 19). The water molecules occupy helical-shaped channels that extend along the *c* direction, running parallel with the Pt…Pt pincer stacks (Fig. 4c). We confirmed that this corresponds to the long axis of the needle-like crystals in Fig. 2f by face-indexing a red Form-I crystal during our X-ray studies (Fig. 5 and Supplementary Fig. 20). These pores provide a clear route for water diffusion, and evidence for continuous diffusion was also observed in diffuse-reflectance measurements performed under controlled humidity (Supplementary Figs. 5–7). In Form-III, the methanol molecules reside in discrete solvent pockets that do not appear to form a continuous channel in the static crystal structure; however, natural lattice breathing motions would be expected to provide a viable route for methanol diffusion along the *c*-axis.

The channel structures explain how the solvent molecules can diffuse out of the structure rapidly during a transition to anhydrous Form-II. No significant void space is present in the Form-II crystal structure, indicating that the voids are filled by the rotation of pincer molecules after solvent evacuation. This suggests that conversion to Form-II would proceed by attachment of solvent molecules at the crystal surface, followed by diffusion into the crystal and progressive conversion to the solvate. Evidence for this is visible in the humidity-controlled reflectance spectroscopy data (Supplementary Figs. 5–7). Under this mechanism, the solvent exchange is diffusion controlled, with the implication that smaller crystallites should reduce the switching time.

The significant structural rearrangements also explain why transitions between the anhydrous and solvate forms in large single crystals could not be achieved without loss of single crystallinity. The rapid solvent diffusion is also facilitated by the fact that the complexes are neutral, and by the absence of counterions in the structures to block the movement of the solvent molecules. This contrasts with Pt- pincer salts such as [Pt(Me_2_bzimpy)Cl][PF_6_], where the [PF_6_]^−^ anions occupy the channels and block solvent movement^4^.

In both Form-I and III, the auxiliary CN^−^ ligands facilitate the key hydrogen-bonding interactions with the solvent. In Form-I, the hydrogen-bonding chains comprise two independent CN…H_2_O interactions with distances of O_3_–N_3_ = 3.027(7) Å and O_3_–N_3_* = 2.921(6) Å, respectively, while in Form-III the single, discrete CN…HOCH_3_ interaction distance is 2.764(5) Å. This implies that the hydrogen bonds are moderate in strength and comprise only a weak electrostatic interaction^44^. That the guest solvent molecules are not tightly bound within the host crystal structure provides further rationale for speed and reversibility of the vapochromic response. From gas-phase quantum-chemical calculations, we estimate hydrogen-bond strengths on the order of 15–40 kJ mol^−1^ per solvent molecule (Supplementary Table 2), which points to relatively weak interactions. These may be compared to the differences in calculated formation energies of Form-I and Form-III compared to Form-II of ~70–80 and 55–75 kJ mol^-1^ per formula unit  (Supplementary Table 3), which suggests that the interaction with the solvent is the dominant energetic driving force for the transformations. The calculations suggest the hydrated form to be more stable than the methanol solvate by ~5–16 kJ mol^−1^, which is consistent with the rapid conversion of Form-II to Form-I in air.

Both the extensive hydrogen-bonding between **1** and its solvent guests, and the steric demands of the surrounding crystal structure, help to rationalise the high selectivity of the vapochromic response. No change is induced in the presence of other sterically-undemanding solvent molecules that contain no hydrogen-bond donor groups (e.g., diethyl ether, acetone or dichloromethane), indicating that the formation of an energetically favourable host-guest hydrogen-bond array is a key driving force for the exclusive uptake of water and methanol. In addition, the size of the available channel space explains why no colour change is observed in **1** on exposure other, bulkier aprotic solvents, e.g., longer-chain alcohols, which might otherwise be expected to induce a vapochromic response. While methanol is small enough to diffuse through the channels, larger alcohols cannot permeate the structure without increasing the Pt…Pt separation beyond the range for significant $d_{z^2}$ overlap. Evidence of this is provided in the observed formation of a yellow ethanol solvate of **1**, which could only be obtained by slow evaporation from ethanol solution. The X-ray structure of the solvate (see Supplementary Figs. 21, 22 and Supplementary Table 4) confirms the disruption of the channel structure and shows that any significant Pt…Pt $d_{z^2}$ overlap is prevented by the steric demands of the ethanol guest.

## Discussion

This study has highlighted the potential of Pt(II)-pincer systems as a platform for vapochromic VOC sensing. The visible response and chemical selectivity are both desirable properties, as is the robust switching over >10^4^ cycles. The subsecond response time is unprecedented for this class of system and further broadens the range of potential applications.

The complex is readily deployed as a smart coating on glass or embedded in a flexible polymer membrane, retaining both visible colour changes and rapid response times. Both architectures comprise microcrystalline needles of the complex, which facilitates the fast diffusion of guest molecules and allows the crystal integrity to be maintained through the stresses induced by the structural changes.

A combination of characterisation techniques has conclusively linked the vapochromic response to the perturbation of intermolecular Pt…Pt interactions in the solid state by the diffusion of guest molecules into and out of the solvent channels. The unusually fast vapochromic response can be explained in terms of the crystal packing arrangement, and indicates that the uptake and release of solvent molecules is diffusion-limited and facilitated by weak interactions with the host crystal structure.

The choice of ancillary ligand L is key to tuning the selectivity and colour response for specific applications: this ligand modulates the energies of the frontier Pt $d$ orbitals in a predictable manner, and provides functional groups in the solvent channels to interact with guest molecules.

Given the nature of the switching mechanism elucidated here, crystal engineering should provide a means to further control the chemical sensitivity and response times with changes in composition, structure and morphology, providing a route to novel vapochromic chemical-sensing devices.

## Methods

### Synthesis

All reactions were carried out under an atmosphere of dry nitrogen. Chloro[methyl 3,5-di(2-pyridyl)benzoato]platinum was prepared according to an adapted literature procedure^35^. 3,5-di(2-pyridyl)benzoate was synthesised by a Stille coupling of methyl 3,5 dibromobenzoate and 2-(tributyl stannyl) pyridine under reflux in toluene at 125 °C for 48 h with catalytic amounts of Pd(PPh_3_)_2_Cl_2_ and LiCl. We also tested an alternative route in which a Negishi coupling between the dibromo starting material and 2-pyridylzinc bromide was carried out in THF at 75 °C for 18 h in the presence of dppf and Pd(dba)_2_. The two procedures afforded the polypyridine ligand in 42 and 60% yield, respectively. The chloride precursor was obtained by refluxing the ligand with K_2_[PtCl_4_] in a 3:1 mixture of acetonitrile and water for 72 h, yielding a vivid yellow solid (55% yield). The chloride complex was then refluxed with AgCN at 85 °C in a 1:1 mixture of acetonitrile and dichloromethane for 24 h. Complex **1** was obtained as a bright red solid following purification of the crude orange/brown suspension and slow recrystallisation from methanol (31% yield; 7/10% across all three steps using the Stille/Negishi coupling routes, respectively, for the ligand preparation). A detailed description of the complete synthesis procedure and characterisation of all intermediates is given in the Supplementary Methods.

### Sample preparation

Thin films of complex **1** were prepared by dissolving it in dichloromethane, drop-casting onto a microscope glass slide and allowing the solvent to evaporate. Similar samples were prepared from chloroform on highly-ordered pyrolytic graphite (HOPG) substrates for scanning electron microscopy, and confirmed to show reversible switching on exposure to dry nitrogen. The microporous polymer PIM-EA-TB was prepared following the reported procedure^40^. To form the membranes, 12 mg of a 10:1 w/w mixture of PIM-EA-TB and complex **1** was dissolved in chloroform (0.5 ml), poured into a 20 mm circular Teflon mould clamped to a 0.25 mm-thick poly(ethylene terephthalate) (PET) film, and the solvent allowed to evaporate over 24 h at room temperature under a chloroform atmosphere. A detailed description of the preparation of the glass coatings and impregnated polymer films is given in Supplementary Methods.

### Characterisation

Complex **1** and the as-prepared Form-I powder were characterised by FT-IR (ATR),^1^H/^13^C NMR, UV/visible and emission spectroscopy, diffuse-reflectance spectroscopy and ESI-MS.

FT-IR (ATR, diamond) (cm^−1^): ν(C≡N) 2118, ν(C = O) 1698. ^1^H NMR (500 MHz, CD_2_Cl_2_) δH (ppm): 9.13 (d,^3^ *J*_H-H_ = 5.2 Hz,^3^ *J*_H-Pt_ = 48.1 Hz, 2 H, ortho-Py), 8.13 (s, 2 H, pincer-Ph), 8.02 (t,^3^ *J*_H-H_ = 7.8 Hz, 2 H, para-Py), 7.80 (d,^3^ *J*_H-H_ = 7.7 Hz, 2 H, meta-Py), 7.28 (t,^3^ *J*_H-H_ = 6.5 Hz, 2 H, meta-Py), 3.91 (s, 3 H, CO_2_CH_3_).^13^C {^1^H} NMR (500 MHz, CD_2_Cl_2_) δC (ppm): 156.17, 140.18, 125.46, 125.19, 120.70, 52.54. Due to the low solubility of the compound, it was not possible to detect signals from quaternary carbons. Mass spectrometry (MeCN): *m*/*z* 536.0977 (proton lost upon ionisation), calc. for [C_19_O_2_N_2_H_12_Pt][MeCN]^−^ 536.0812. The expected isotope pattern was observed. In DSC measurements (10 °C min^−1^), the onset of decomposition was observed at 301 °C.

Solid state UV-visible spectra were recorded using a microspectrophotometer with a 50 µm diameter probe beam, employing mirrored lenses (Bruker) mounted in an off-axis geometry and a deuterium-halogen lamp as the light source (Ocean Optics), with absorption being monitored over a 200–750 nm wavelength range using a Shamrock 303 imaging spectrograph (Andor). Time-resolved measurements, were recorded every 100 ms for the duration of the experiment.

Diffuse-reflectance spectra were collected using an Ocean Optics Maya 2000 Pro (220–1100 nm spectral range) with a DL-2000-BAL deuterium-tungsten light source and QP600–2-SR-BX 600 μm solarisation-resistant fibres, calibrated against a WS-1-SL spectralon diffuse-reflectance standard.

A detailed description of the setup used to quantify the sensitivity of the films to water vapour and the cycling endurance can be found in the Supplementary Methods.

### Crystallography

Single-crystal X-ray diffraction data were collected on Beamline 11.3.1 at the Advanced Light Source, Berkeley, CA, using a Bruker AXS D8 diffractometer equipped with an APEX II CCD detector. The temperature was controlled using an Oxford Cryosystems Cryostream Plus. Data collection and processing were carried out using the Bruker APEX II software. The structures were solved by dual-space methods using SHELXT-2014^45^ and refined by full-matrix least-squares on *F* ^2^ using SHELXL-2014^46^. A detailed description of the sample preparation and X-ray measurements, together with key crystallographic data, may be found in Supplementary Methods, Supplementary Figs. 10–12 and 20–22, and Supplementary Tables 1 and 4.

### Quantum-chemical modelling

Calculations were performed within the density-functional theory (DFT) formalism. Molecular modelling was carried out using the Gaussian 09 suite of programs^47^, using the PBE0 exchange-correlation functional^48^. A basis of 6–31 g(d) quality was used for the main-group atoms^49^, and Pt, Br and I were treated using the LANL2DZ effective-core pseudopotential^50^ and corresponding double-zeta basis set. Initial models of various Pt–pincer complexes were built using Avogadro^51^, optimised, and confirmed to be energetic minima by computing the vibrational frequencies. Natural population analyses (NPAs) were carried out on the optimised structures to obtain the energies of the $d_{z^2}$ orbitals^52^. Calculations were also carried out on species extracted from the X-ray structures to estimate gas-phase interaction energies. Solid-state calculations were carried out within the pseudopotential plane-wave DFT formalism implemented in the Vienna *ab initio* simulation package (VASP)^53^ and Quantum ESPRESSO (QE)^54^ codes. Initial coordinates were taken from the X-ray structures and optimised in VASP using the PBEsol functional^55^. Projector augmented-wave (PAW) pseudopotentials^56^ were used to model the ion cores, the electronic structures were expanded in a plane-wave basis with a 750 eV kinetic-energy cutoff, and the electronic Brillouin zones were sampled using a uniform Γ-centred k-point mesh with 1 × 1 × 3 subdivisions. Additional calculations were carried out on 1 × 1 × 3 supercell expansions of the optimised structures in QE using the same functional, and comparable PAW pseudopotentials and convergence criteria, to visualise the frontier wavefunctions. Calculations were also carried out on isolated molecules and complexes extracted from the crystal structures to obtain formation energies. The absorption spectra of the optimised crystal structures were modelled using the linear-optics routines in VASP^57^ with PBE0. A subset of the molecular and periodic calculations were performed with the DFT-D3 dispersion correction with the Becke–Johnson damping function^58, 59^ applied to the respective base exchange-correlation functionals. More detailed information on the computational modelling performed in this study is given in Supplementary Note 1.

### Data availability

The X-ray crystallographic coordinates for the structures reported in this study have been deposited at the Cambridge Crystallographic Data Centre (CCDC), under deposition numbers 954459, 954461, 1493352 and 1577640. These data can be obtained free of charge from the Cambridge Crystallographic Data Centre at http://www.ccdc.cam.uk/data_request/cif. The data from the computational modelling, including optimised structures and simulated absorption spectra, are available online at https://doi.org/10.15125/BATH-00440. The computer code used to analyse the diffuse-reflectance spectra is available in a public GitHub repository at https://github.com/JMSkelton/MayaPy.

## Electronic supplementary material

Supplementary Information

Peer Review File

Description of Additional Supplementary Files

Supplementary Movie 1

Supplementary Movie 2

Supplementary Movie 3

Supplementary Movie 4

Supplementary Movie 5
