# Supplementary material for: A rapidly-reversible absorptive and emissive vapochromic Pt(II) pincer-based chemical sensor
Source: Nat Commun. 2017 Nov 27;8:1800. doi: 10.1038/s41467-017-01941-2 (PMC5702612; doi:10.1038/s41467-017-01941-2)
Supplement: Supplementary file 1 — Supplementary Information [file 41467_2017_1941_MOESM1_ESM.pdf]

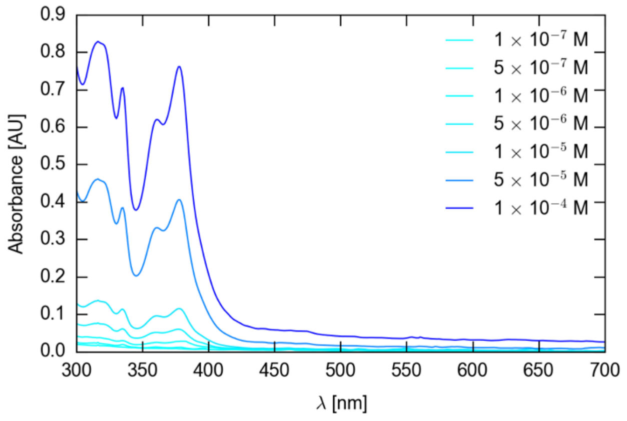

Supplementary Figure 1 Solution absorption spectra of the Pt-pincer complex **1** in 10^{-7}-10^{-4} M CH_2Cl_2 solutions.

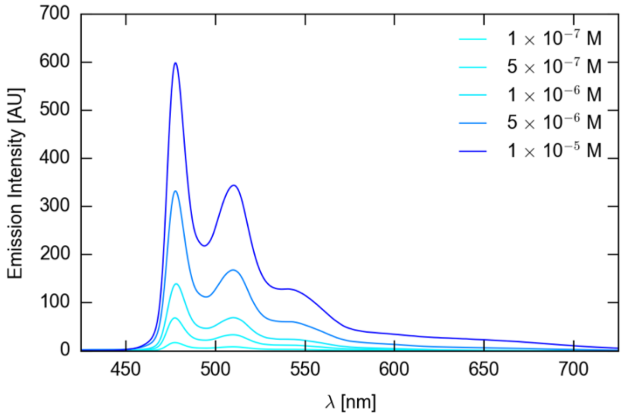

Supplementary Figure 2 Solution emission spectra of the Pt-pincer complex **1** in 10^{-7}-10^{-4} M CH_2Cl_2 solutions (λ_{Ex} = 395 nm).

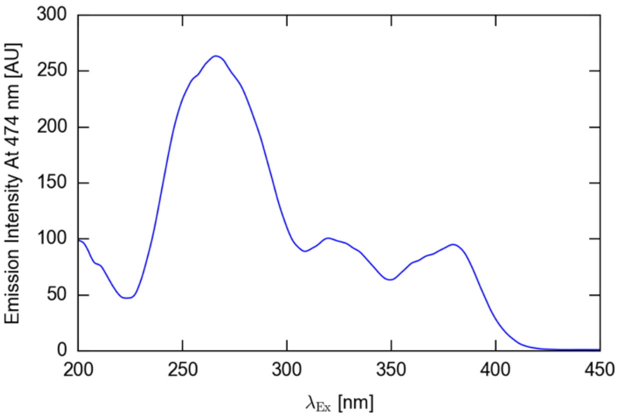

Supplementary Figure 3 Emissive response of the Pt-pincer complex **1** at 474 nm as a function of excitation wavelength in a 10^{-6} M CH_2Cl_2 solution.

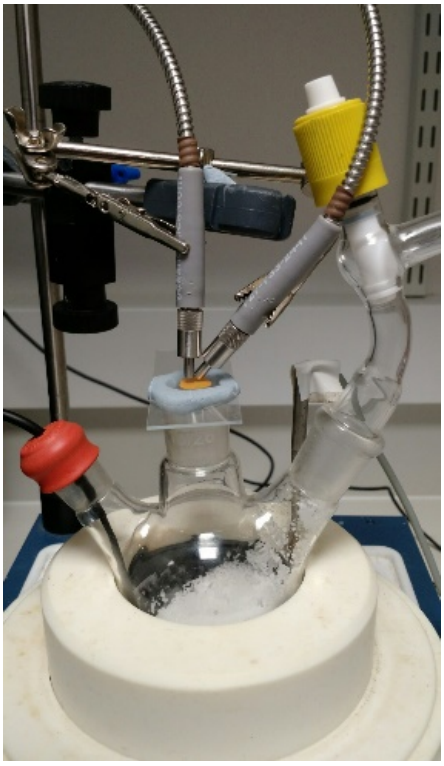

Supplementary Figure 4 Photograph of the setup used to determine the sensitivity of the films of complex **1** coated on glass to atmospheric water vapour. In a sealed three-neck round-bottom flash, a film of **1**, initially in the anhydrous Form-II phase, is held under nitrogen gas at atmospheric pressure (central neck). The flask contains a LiCl(sq) slurry and is kept in a Polar Bear Plus reactor to allow the water vapour concentration to be controlled (bottom). A thermocouple is inserted through a rubber seal to allow the temperature of the solution to be monitored (left neck). A light source and reflectance probe are positioned at 45 ° to record reflectance spectra through the supporting glass slide (top).

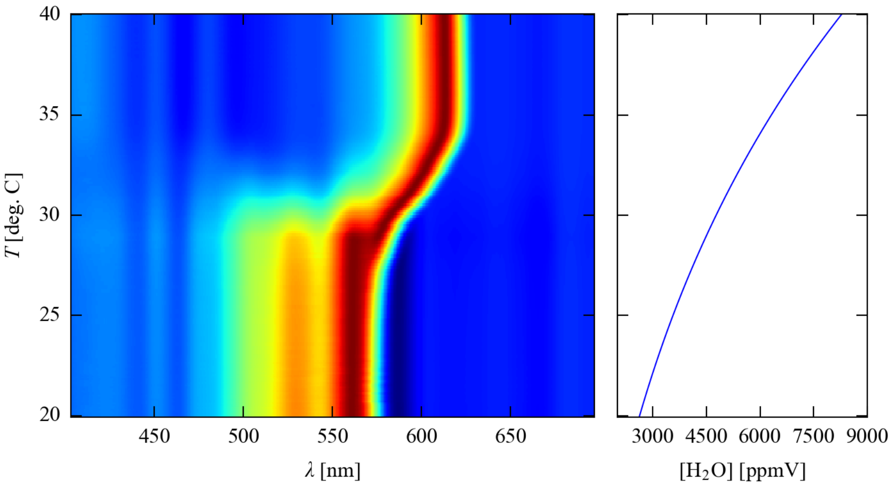

Supplementary Figure 5 Evolution of the first-derivative diffuse-reflectance spectrum of a film of complex **1** as a function of the solution temperature. The right-hand panel relates the temperature to the atmospheric water-vapour concentration.

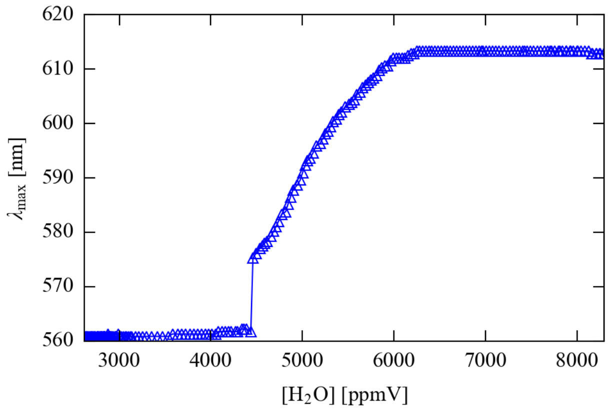

Supplementary Figure 6 Position of the maxima in the first-derivative spectra used to build the plot in Supplementary Figure 5 as a function of the water-vapour concentration, showing the range of concentrations over which the Form-II → Form-I transition occurs.

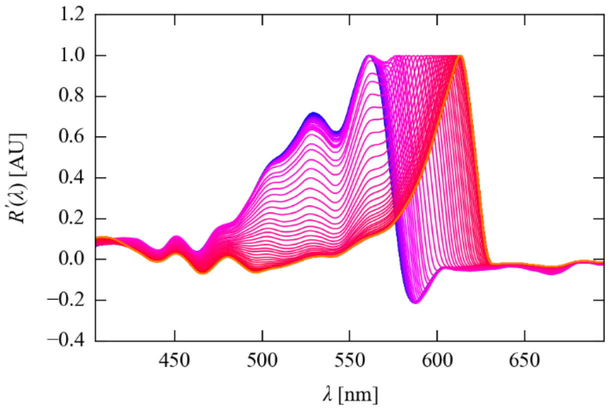

Supplementary Figure 7 A subset of the spectra from the 2D plot in Supplementary Figure 5. The spectra are colour-coded by the solution temperature at which they were collected, with blue and orange corresponding to low and high temperature (i.e. low and high water-vapour concentrations), respectively. Features from the colour-transition edge of the yellow Form-II occur at ~500, 530 and 560 nm, whereas the red/orange Form-I edge produces a single peak between approx. 570 and 610 nm.

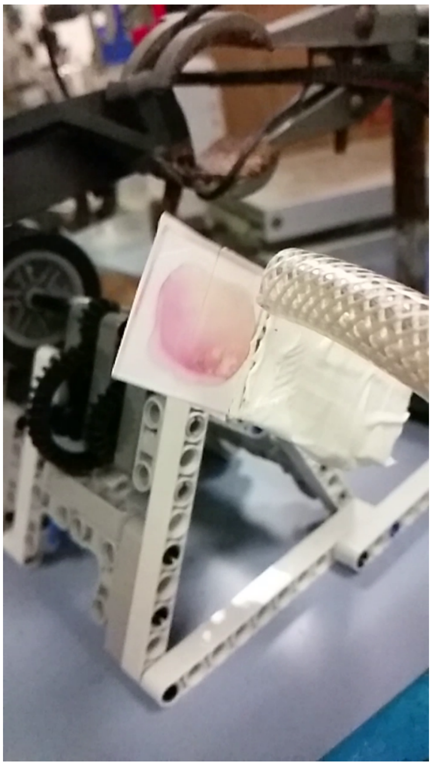

Supplementary Figure 8 Setup used to test the durability of the vapochromic response of complex **1**. A film of **1** coated onto glass is attached to a spinner operated at 0.6 Hz, and passes through a dry gas stream once per cycle. The image shows the sample film partly under the gas flow, such that the colour change extends over approx. half the film area.

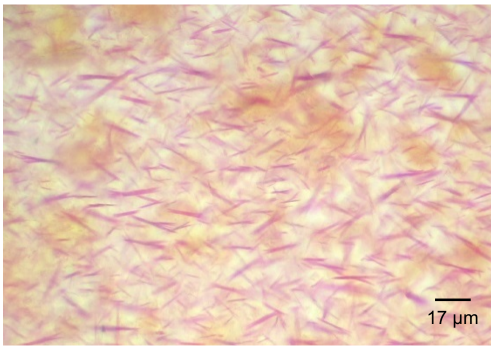

Supplementary Figure 9 Optical micrograph of a flexible polymer membrane impregnated with microcrystals of complex **1**.

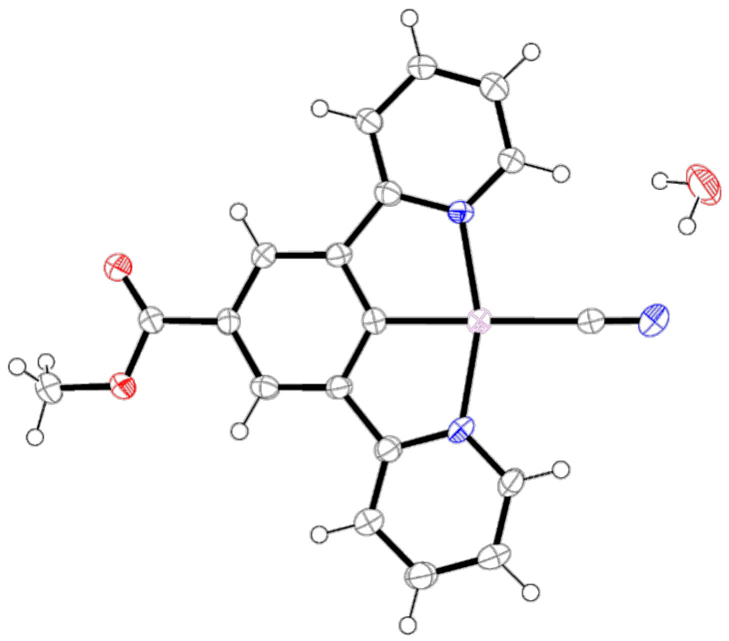

Supplementary Figure 10 Atomic arrangement in the asymmetric unit of the solved single-crystal X-ray structure of Form-I (hydrated, red).

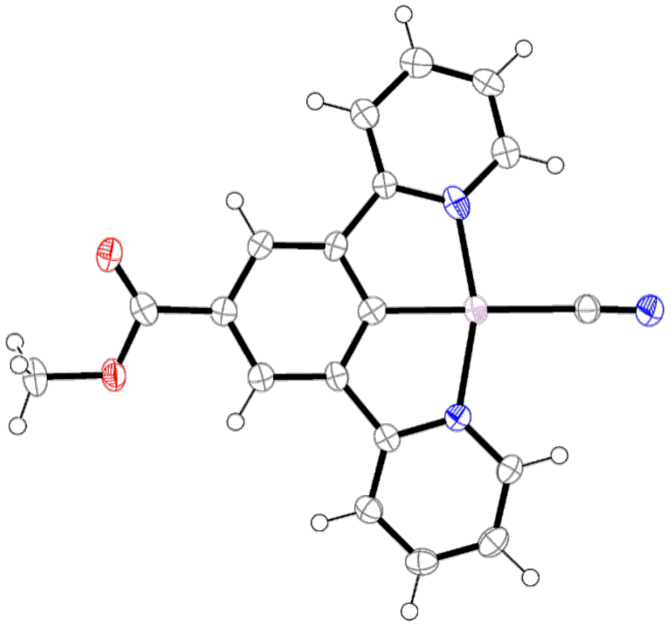

Supplementary Figure 11 Atomic arrangement in the asymmetric unit of the solved single-crystal X-ray structure of Form-II (anhydrous, yellow).

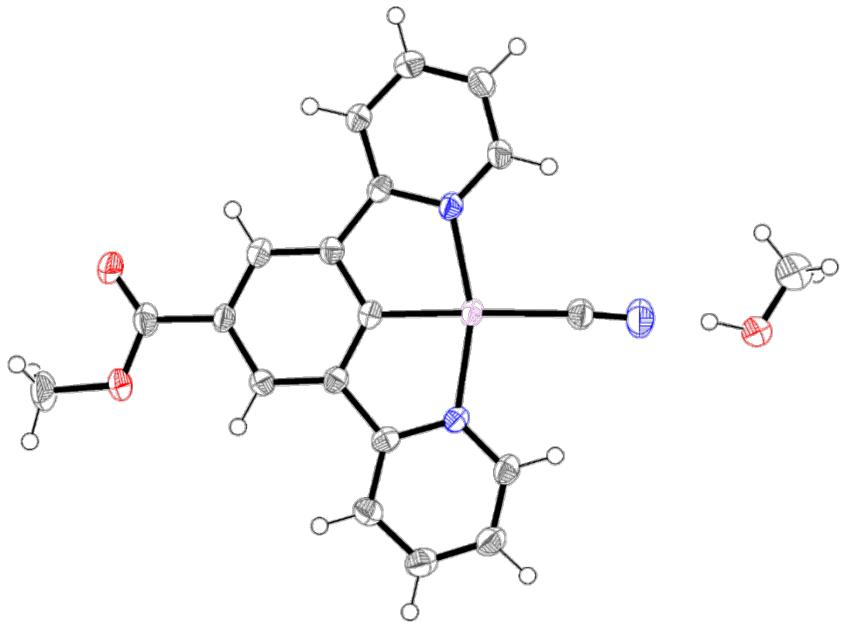

Supplementary Figure 12 Atomic arrangement in the asymmetric unit of the solved single-crystal X-ray structure of Form-III (methanolic, blue).

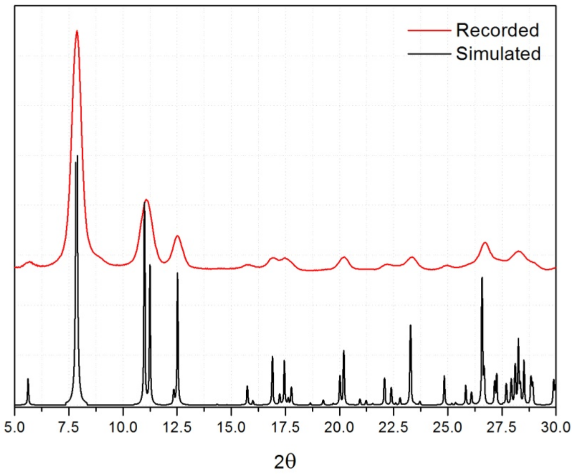

Supplementary Figure 13 Comparison of the powder X-ray diffraction (PXRD) pattern simulated from the single-crystal X-ray structure of Form-I to the pattern recorded from a bulk powder sample.

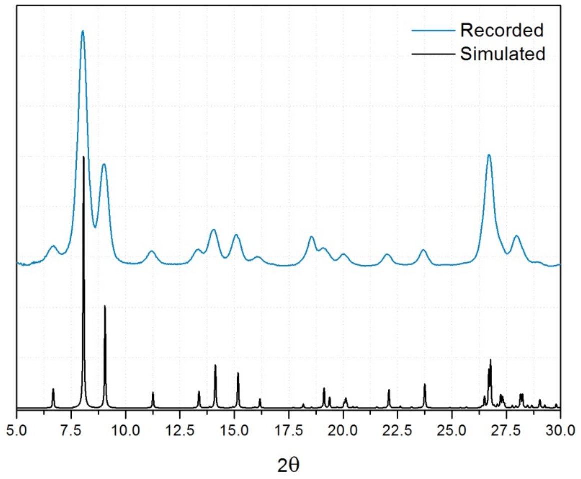

Supplementary Figure 14 Comparison of the powder X-ray diffraction (PXRD) pattern simulated from the single-crystal X-ray structure of Form-III to the pattern recorded from a bulk powder sample.

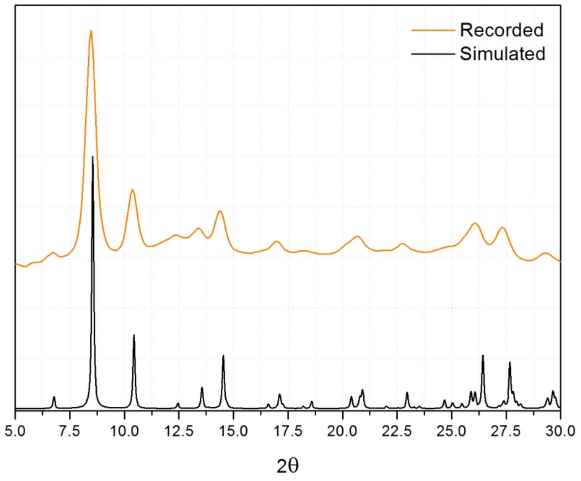

Supplementary Figure 15 Comparison of the powder X-ray diffraction (PXRD) pattern simulated from the single-crystal X-ray structure of Form-II to the pattern recorded from a bulk powder sample.

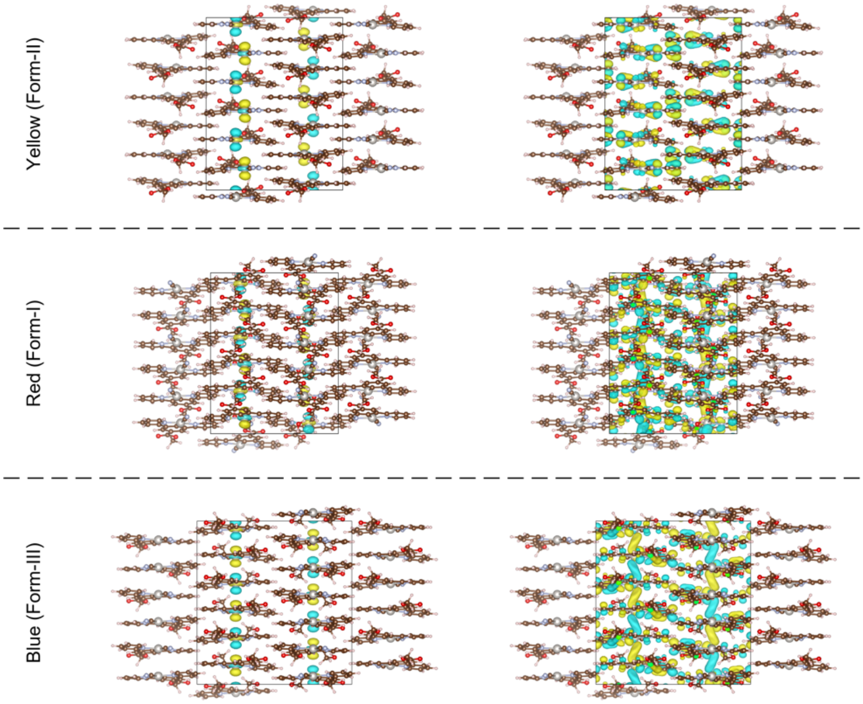

Supplementary Figure 16 Highest-occupied (left) and lowest-unoccupied (right) Kohn-Sham wavefunctions at the electronic Brillouin zone centre (Γ-point) of 1×1×3 supercell expansions of the optimised crystal structures. Top: anhydrous Form-II (yellow); Centre: hydrated Form-I (red); Bottom: methanolic Form-III (blue). The images in the left-hand column are the same as those in Fig. 4 (d) in the text.

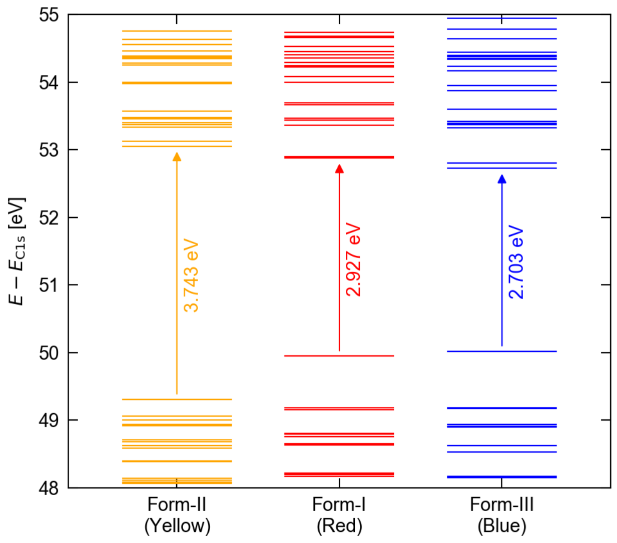

Supplementary Figure 17 Calculated Γ-point Kohn-Sham orbital energies in the anhydrous Form-II (yellow), hydrated Form-I (red) and methanolic Form-III (blue) structures. The energy gaps between the highest-occupied and lowest-unoccupied crystal orbitals (HOCOs/LUCOs) are indicated by arrows, with the size of the gaps as marked.

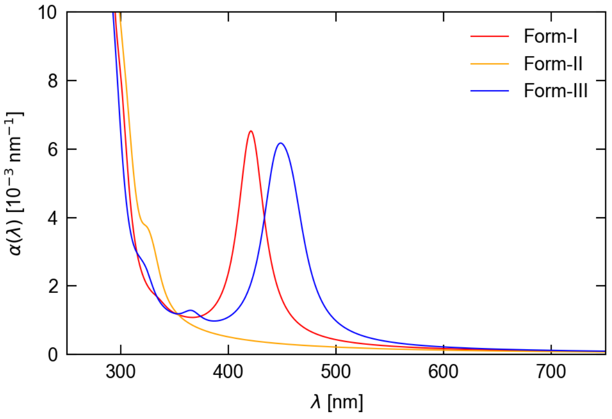

Supplementary Figure 18 Simulated wavelength-dependent absorption coefficient (α) of the optimised hydrated Form-I (red), anhydrous Form-II (yellow) and methanolic Form-III (blue) structures.

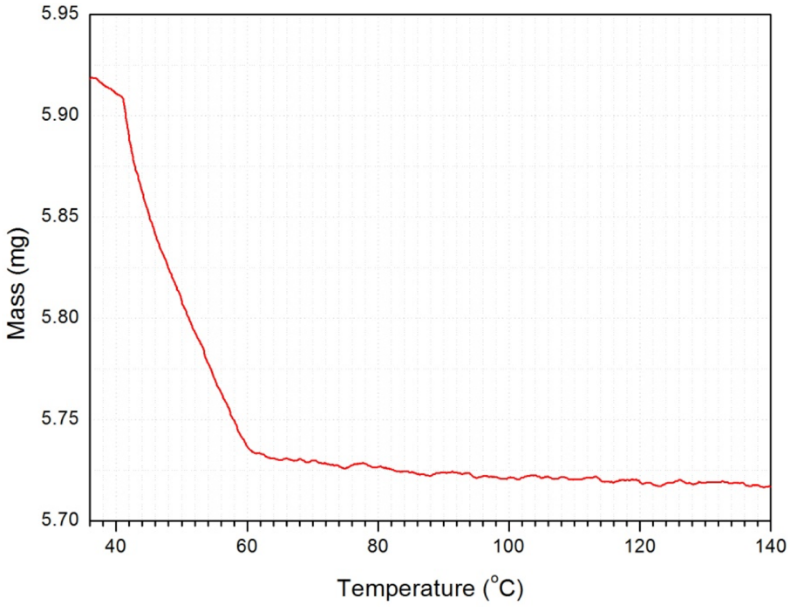

Supplementary Figure 19 Thermogravimetric analysis (TGA) of Form-I. The mass loss up to ~60 °C corresponds to around 17 g mol^{-1}, confirming the presence of one molecule of water per molecule of **1** as observed crystallographically.

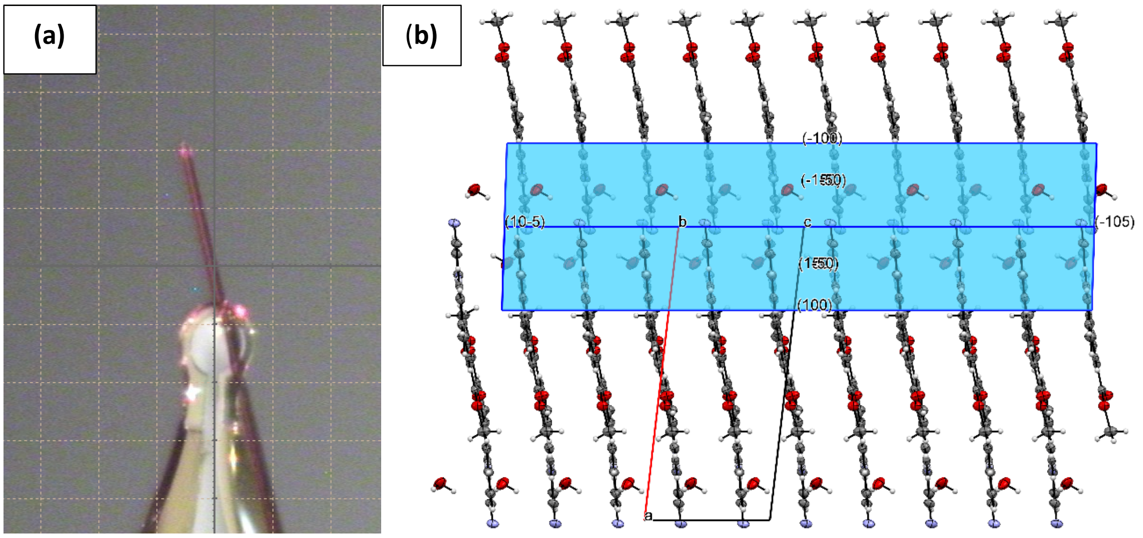

Supplementary Figure 20: (a) Red needle crystal of Form I used for crystal face indexing with the Rigaku-Oxford Diffraction software CrysAlis Pro.[1] (b) Alternative view of Fig. 5 in the text, showing the reconstruction of the natural crystal faces determined by face indexing, superimposed on the crystal-packing diagram of Form I, viewed along the crystallographic *b* axis (*c* axis horizontal).

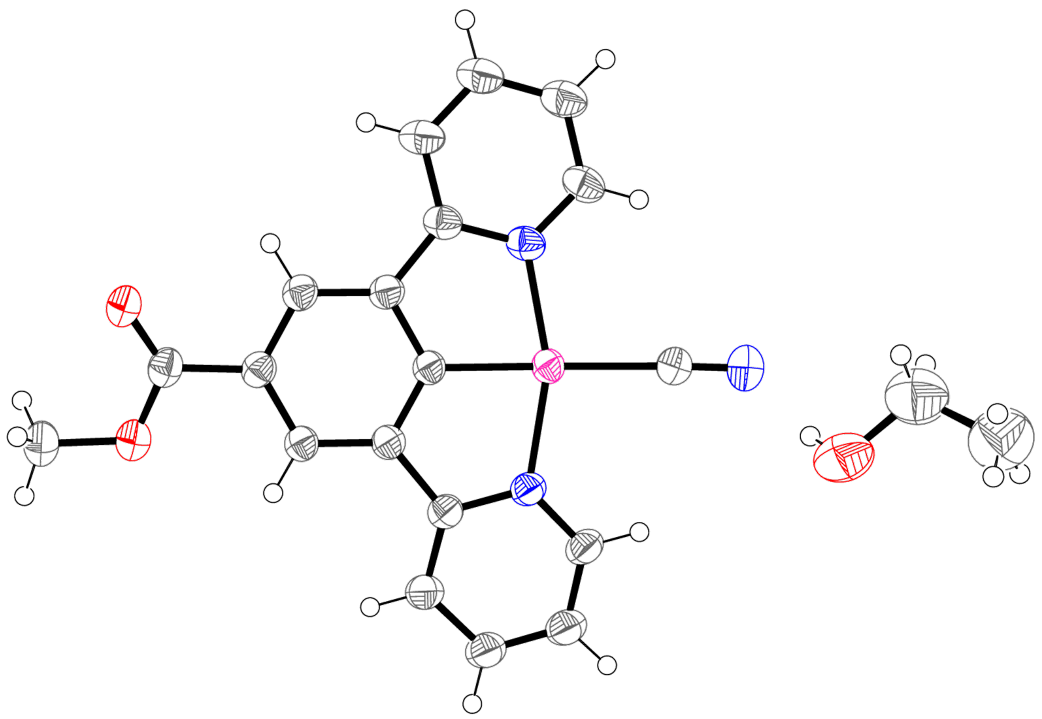

Supplementary Figure 21 Atomic arrangement in the asymmetric unit of the solved single-crystal X-ray structure of **1**.EtOH (ethanol solvate, yellow), ellipsoids at 50% probability for non-hydrogen atoms.

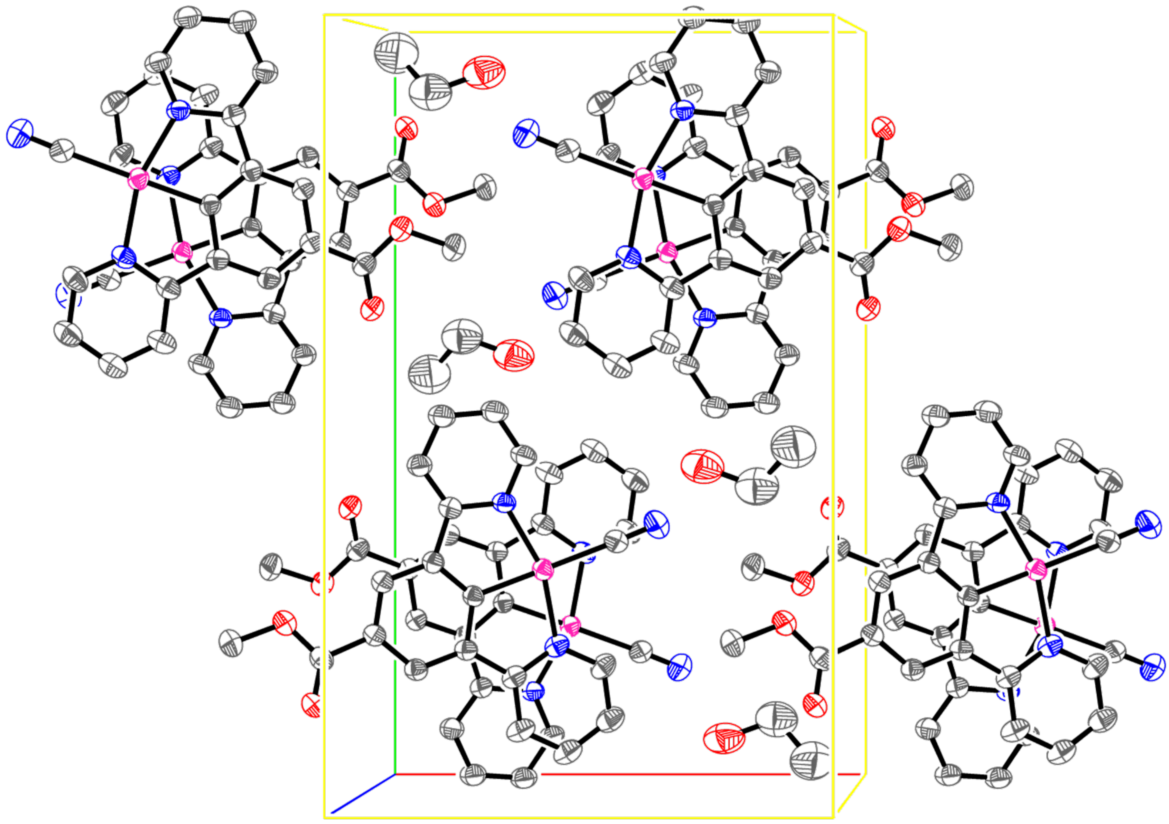

Supplementary Figure 22 Crystal packing arrangement for 1.EtOH, viewed normal to the [001] direction, ellipsoids at 50% probability and hydrogen atoms removed for clarity.

Supplementary Table 1 Crystallographic data for the single-crystal X-ray structures of Form-I, Form-II and Form-III.

	Form-I	Form-II	Form-III
Empirical formula	$C_{19}H_{15}N_3O_3Pt$	$C_{19}H_{13}N_3O_2Pt$	$C_{20}H_{17}N_3O_3Pt$
Formula weight [g mol^{-1}]	528.43	510.41	542.45
T [K]	150(2)	150(2)	150(2)
Crystal system	Monoclinic	Monoclinic	Monoclinic
Space group	$P2_1/c$	$P2_1/c$	$P2_1/c$
a [Å]	15.848(13)	13.166(3)	13.274(2)
b [Å]	16.096(14)	16.941(4)	19.515(3)
c [Å]	6.729(6)	6.9375(18)	6.7743(12)
α [°]	90	90	90
β [°]	97.018(15)	97.627(3)	94.642(3)
γ [°]	90	90	90
V [Å^3]	1704(3)	1533.7(6)	1749.1(5)
Z	4	4	4
ρ_{calc} [g cm^{-3}]	2.060	2.211	2.060
μ / mm^{-1}	10.233	11.359	9.972
F(000)	1008	968	1040
Crystal size [mm]	0.03 × 0.01 × 0.01	0.02 × 0.01 × 0.01	0.02 × 0.01 × 0.01
Radiation	Synchrotron (λ = 0.7749 Å)	Synchrotron (λ = 0.7749 Å)	Synchrotron (λ = 0.7749 Å)
2θ range for data collection [°]	6.2 - 69.146	6.252 - 67.454	5.656 - 69.328
Index ranges	$-22 \le h \le 22$, $-23 \le k \le 23$, $-9 \le l \le 9$	$-18 \le h \le 18$, $0 \le k \le 24$, $0 \le l \le 9$	$-18 \le h \le 19$, $-28 \le k \le 28$, $-9 \le l \le 9$
Reflections collected	18719	4685	19384
Independent reflections	5300 [R_{int} = 0.0465, R_{sigma} = 0.0464]	4685 [R_{int} = 0.0502, R_{sigma} = 0.0473]	5532 [R_{int} = 0.0384, R_{sigma} = 0.0368]
Data / restraints / parameters	5300 / 2 / 242	4685 / 0 / 228	5532 / 3 / 250
Goodness-of-fit on F^2	0.946	1.099	0.987
Final R indexes [I>=2σ(I)]	R1 = 0.0296, wR2 = 0.0588	R1 = 0.0383, wR2 = 0.0892	R1 = 0.0255, wR2 = 0.0511
Final R indexes [all data]	R1 = 0.0509, wR2 = 0.0639	R1 = 0.0494, wR2 = 0.0928	R1 = 0.0576, wR2 = 0.0585
Largest diff. peak/hole [e Å^{-3}]	1.52 / -1.43	1.84 / -3.31	0.74 / -1.47

Supplementary Table 2 Gas-phase formation energies of the Form-I, Form-II and Form-III complexes, calculated using the Vienna *ab initio* simulation package[2] with the PBEsol functional[3] and PBEsol with the DFT-D3 dispersion correction with Becke-Johnson damping (PBEsol + D3 (BJ)).[4, 5] These complexation energies may be compared to the solid-state formation energies in Supplementary Table 3. For comparison, energies obtained with the dispersion-corrected PBE0 functional[6] (PBE0 + D3 (BJ)) using Gaussian 09[7] are also given.

	E_F [eV]	$E_{F,\mathbf{Form\text{-}II}}$ [kJ mol^{-1} per F.U.]
	PBEsol	
**Form-I**	1.20	-47.3
**Form-II**	0.21	-
**Form-III**	1.31	-52.6
	PBEsol + D3 (BJ)	
**Form-I**	2.27	-52.7
**Form-II**	1.17	-
**Form-III**	2.33	-55.8
	PBE0 + D3 (BJ)	
**Form-I**	3.10	-76.4
**Form-II**	1.50	-
**Form-III**	3.04	-73.6

Supplementary Table 3 Solid-state formation energies of the hydrated Form-I, anhydrous Form-II and methanolic Form-III, calculated relative to the gas-phase components using the PBEsol functional[3] with and without the DFT-D3 dispersion correction with Becke-Johnson damping (PBEsol + D3 (BJ)).[4, 5] The second column shows the per formula unit (F.U.) differences in the formation energies of the two solvated forms relative to the anhydrous Form-II.

	E_F [eV]	$E_{F,\mathbf{Form\text{-}II}}$ [kJ mol^{-1} per F.U.]
	PBEsol	
**Form-I**	5.58	-72.0
**Form-II**	2.58	-
**Form-III**	4.20	-56.0
	PBEsol + D3 (BJ)	
**Form-I**	12.70	-78.3
**Form-II**	9.43	-
**Form-III**	12.45	-72.3

Supplementary Table 4 Crystallographic data for the single-crystal X-ray structure of the ethanol solvate form of complex **1**, **1**.EtOH.

	**1**.EtOH
Empirical formula	$C_{21}H_{19}N_3O_3Pt$
Formula weight [g mol^{-1}]	556.48
T [K]	150(2)
Crystal system	Monoclinic
Space group	$P2_1/c$
a [Å]	12.8860(7)
b [Å]	20.3320(10)
c [Å]	7.2494(4)
α [°]	90
β [°]	99.104(5)
γ [°]	90
V [Å^3]	1875.40(17)
Z	4
ρ_{calc} [g cm^{-3}]	1.971
μ / mm^{-1}	14.233
F(000)	1072
Crystal size [mm]	0.179 × 0.017 × 0.007
Radiation	CuKα (λ = 1.54184)
2θ range for data collection [°]	6.948 - 131.634
Index ranges	$-15 \leq h \leq 15$, $-24 \leq k \leq 23$, $-8 \leq l \leq 8$
Reflections collected	15576
Independent reflections	3261 [R_{int} = 0.0660, R_{sigma} = 0.0399]
Data / restraints / parameters	3261 / 182 / 255
Goodness-of-fit on F^2	1.021
Final R indexes [I>=2σ(I)]	R_1 = 0.0437, wR_2 = 0.1121
Final R indexes [all data]	R_1 = 0.0519, wR_2 = 0.1211
Largest diff. peak/hole [e Å^{-3}]	1.98 / -0.94

Supplementary Note 1

In this note, we discuss in detail the procedure and results from the computational modelling performed to support the experimental characterisation in this study.

All quantum-chemical calculations were performed within the Kohn-Sham density-functional theory formalism,[8, 9] with molecular and periodic calculations carried out as described below.

Molecular calculations

Molecular modelling was performed to compare the d_{z^2} orbital energies of a series of square-planar Pt-pincer complexes with the methyl 3,5-di(2-pyridyl)benzoato pincer and a range of monoanionic ancillary ligands, *viz.* I^-, Br^-, SCN^-, Cl^-, N_3^-, F^-, NCO^-, OH^-, NCS^-, NO_2^-, and CN^-.

Initial models were build using the Avogadro software,[10] and the geometries were optimised using the Gaussian 09 suite of programs[7] with the PBE0 exchange-correlation functional[6] and the default convergence criteria. A basis of 6-31g(d) quality was used for the main-group atoms,[11] and the Pt, Br and I atoms were treated using the LANL2DZ effective-core pseudopotential[12, 13] and corresponding double-zeta basis set. The optimised structures were confirmed to be energetic minima by the absence of negative vibrational modes in the nuclear Hessian matrices. Natural population analyses (NPAs)[14] were performed on the optimised structures to obtain the energies of the d_{z^2} orbitals, which were then compared to the energies of the frontier molecular orbitals (Fig. 1 in the text).

We also performed a series of calculations to estimate the complexation energies associated with the Pt-pincer dimer (Form-II structure) and the dimer with H-bonded water (Form-I) and methanol (Form-III). Initial models were built by extracting the complexes and the constituent components, *viz.* the Pt-pincer complex **1**, H_2O, methanol, and the (Pt-pincer)$_2$, (Pt-pincer.H_2O)$_2$ and (Pt-pincer.CH_3OH)$_2$ dimers from the experimental X-ray structures. These were then optimised with the same parameters as for the initial ligand comparison. This set of calculations was performed with the DFT-D3 dispersion correction[4] with Becke-Johnson damping[5] added to the PBE0 exchange-correlation functional.

Periodic calculations

Periodic calculations were carried out within the pseudopotential plane-wave DFT formalism implemented in the Vienna *ab initio* simulation package (VASP)[2] and Quantum ESPRESSO (QE)[15] codes.

Initial coordinates for the anhydrous, hydrated and methanolic forms of **1** (i.e. Form-II, Form-I and Form-III) were taken from the X-ray structures and fully optimised in VASP until the magnitude of the forces on the ions fell below 10^{-2} eV Å^{-1}. The PBEsol functional[3] was used to model quantum-mechanical exchange and correlation, with and without the D3 dispersion correction with Becke-Johnson damping[4, 5] (we denote the dispersion-corrected functional by PBEsol + D3 (BJ)). Projector augmented-wave (PAW) pseudopotentials[16, 17] treating the C, O and N 2s and 2p and the Pt 6s and 5d states as valence were used to model the ion cores. The electronic structure was expanded in a plane-wave basis with a 750 eV kinetic-energy cutoff, and the electronic Brillouin zone was sampled using a Γ-centered Monkhorst-Pack *k*-point mesh[18] with 1×1×3 subdivisions. The precision of the charge-density grids was chosen automatically to avoid aliasing errors. These convergence criteria were found to be sufficient to converge the absolute values of the total energy and stress tensor to within 1 meV per atom and 1 kbar (0.1 GPa), respectively. During the wavefunction optimisation, the tolerance for the electronic minimisation was set to 10^{-8} eV, and the PAW projection was performed in real space.

The optimised lattice parameters were found to be in good agreement with the experimental structures. The PBEsol calculations consistently overestimated the length of the *c* axis by ~1.5-3.5 %, generally leading to expanded cell volumes, whereas the dispersion-corrected calculations showed the reverse trend, yielding optimised cells with contracted cell volumes and *c*-axis lengths around 2.5-4.5 % shorter than those in the X-ray structures. This suggests that the dispersion-corrected functional yields a better description of the π-stacking interaction between adjacent Pt-pincer molecules, but perhaps overestimates the magnitude of the attractive dispersion force. The lengths of the *a* and *b* axes of the PBEsol-optimised structures were within 0.5 % of the experimental values, whereas the PBEsol + D3 (BJ) calculations show larger deviations of up to 1.5 %, and both sets of calculations show roughly equal discrepancies in the β angle, which range from ~0.1-1.5 %. Overall, the calculations suggest that the "bare" PBEsol calculations yield structures closer on average to those measured by X-ray diffraction, and, crucially, these calculations also better reproduce the measured Pt-Pt distances in the experimental structures. We therefore opted to use PBEsol as the basis for our more detailed electronic-structure analysis.

While VASP has routines for visualising the electron density associated with the Kohn-Sham wavefunctions, it does not have a straightforward means of visualising the phase, which is important in the present study for determining the nature of the orbitals. In QE, the phases of the wavefunctions at the Brillouin-zone centre (Γ) can be visualised using the PostProc program. Additional calculations were therefore carried out on 1×1×3 supercell expansions of the optimised structures, for which sampling the electronic structure at the Brillouin-zone centre (Γ point) is appropriate, using the PWscf program to compute the Kohn-Sham wavefunctions and PostProc to visualise the frontier electronic states.

For consistency with VASP, we used PBEsol with PAW pseudopotentials,[1] generated using the same technique[17] and with the same valence and core electrons as those in the VASP calculations, and applied comparable convergence criteria, *viz.* a plane-wave cutoff of 55 Ry for the electronic wavefunctions and a tolerance of 10^{-9} Ry for the wavefunction minimisation.

A single electronic minimisation on the relaxed (single) unit cells, using the same *k*-point mesh as in the VASP calculations, yielded electronic gaps between the highest-occupied and lowest-unoccupied crystal orbitals (HOCOs/LUCOs) within 1 %, with a maximum offset of 8 meV, providing some assurance that the two codes produce similar electronic structures.

To further assess the correspondence between the VASP and QE calculations, we allowed the relaxed unit cells obtained from the PBEsol calculations in VASP to optimise in QE, again using an identical *k*-point mesh and a similar force-convergence criterion of 5×10^{-4} a.u. We then compared the differences in the optimised lattice parameters, the maximum and average absolute deviation in atomic positions x, and the direct HOCO-LUCO bandgaps at both the irreducible *k*-points in the sampling mesh obtained from the two sets of calculations.

Across the three relaxations, there were negligible (< 0.5 %) changes in the lengths of the crystallographic a and b axes, and the maximum and average absolute changes in atomic positions were below 0.2 and 0.1 Å, respectively. On the other hand, all three calculations produced a 1-2 % lengthening of the c axes, leading to a 1-3 % increase in the cell volumes, and the re-optimisation of the Form-I cell also led to a 1.1 % change in the β angle. Since the electronic structure is particularly sensitive to the length of the c axis (i.e. the Pt-Pt distance), these changes led to changes in the calculated PBEsol bandgap of ~50-100 meV (2.5 - 9 %) compared to the VASP calculations. While some of these changes are less than satisfactory, given the differences between the software and technical setups - in particular, the inevitable differences in the pseudopotentials, despite their being generated using the same technique - we consider them acceptable. We should also note that the differences may be due in part to the two codes using different break criterion for the cell relaxation, despite our attempting to match convergence parameters as closely as possible.

Based on these calculations, we are confident that using QE to visualise the frontier orbitals of the VASP-optimised structures is reasonable in this case, despite differences between the two codes.

[1] We used the H.pbesol-kjpaw_psl.0.1.UPF, C.pbesol-n-kjpaw_psl.0.1.UPF, N.pbesol-n-kjpaw_psl.0.1.UPF, O.pbesol-n-kjpaw_psl.0.1.UPF and Pt.pbesol-n-kjpaw_psl.0.1.UPF potentials, available from the Quantum ESPRESSO pseudopotential library at http://www.quantum-espresso.org/pseudopotentials/.

Electronic-structure analysis

The HOCOs and LUCOs obtained from the QE supercell calculations are illustrated in Supplementary Figure 16**.** As discussed in the main text, this analysis shows that the HOCOs comprise an antiphase interaction between the Pt d_{z^2} orbitals on adjacent Pt centres, while the LUCOs are based on the pincer ligands with an in-phase interaction between adjacent molecules.

More accurate electronic-structure calculations were carried out by performing single-point calculations on the optimised structures in VASP using PBE0,[6] from which we simulated solid-state absorption spectra by calculating the frequency-dependent dielectric functions with the linear-optics routines in VASP.[19] In these calculations, a more accurate reciprocal-space PAW projection was employed, and the number of electronic bands was increased to roughly triple the default to ensure convergence of the sum over empty Kohn-Sham states.

From the spectrum of energy levels at the Γ point in the three structures (Supplementary Figure 17), where the smallest HOCO-LUCO gap occurs in each case, the effects of the structural changes between Form-II, Form-I and Form-III can be seen to raise the energy of the HOCO and to lower the energy of the LUCO, thereby resulting in a substantial reduction of the HOCO-LUCO gap. We obtain bandgaps of 3.74 eV (331 nm), 2.93 eV (424 nm) and 2.70 eV (459 nm) for the yellow Form-II, red Form-I and blue Form-III, respectively, which is consistent with the ordering of the reflected colours.

The link between the changes in the frontier orbitals and HOCO-LUCO gaps and the shift in absorption maxima is confirmed by the simulated absorption spectra of the three structures (Supplementary Figure 18). Although the approximations inherent in this implementation of time-dependent density functional theory (TD-DFT) are not expected accurately to reproduce the experimental absorption spectra,[20] the qualitative spectral shifts between the yellow Form-II, red Form-I and blue Form-III are correctly reproduced by these calculations, taking into account the normalisation of the spectra in Fig. 3 (b) in the text.

Formation and complexation energies

To explore the energetics of the transformations between Form-I, Form-II and Form-III, we calculated and compared solid-state formation energies relative to the gas-phase components (Supplementary Table 3). The reference gas-phase calculations were performed by extracting relevant species from the X-ray structures and placing them at the centre of a large cubic cell with an (initial) distance of ~15 Å between periodic images. The atomic positions (but not the cells) were then optimised using the same tolerances as in the periodic calculations, with the electronic wavefunctions modelled at the Γ point.

These calculations establish a stability order of Form-I > Form-III > Form-I, which is consistent with the fact that the anhydrous form readily takes up methanol and water, and that the methanolic Form-III converts to the hydrated Form-I in air. The dispersion-corrected PBEsol + D3 (BJ) calculations predict substantially higher formation energies than the "bare" PBEsol ones, which reinforces the conclusion that the former more accurately captures the weak π-stacking interaction between the Pt-pincer complexes in the solid state. PBEsol predicts Form-III and Form-I to be 56 and 72 kJ mol^{-1} per formula unit (F.U.) more stable than the anhydrous form, and thus the hydrated form to be 16 kJ mol^{-1} per F.U. more stable than the methanol solvate. The dispersion correction predicts a considerably smaller difference of 6.0 kJ mol^{-1} per F.U., with both solvates being 70-80 kJ mol^{-1} per F.U. more stable than the anhydrous form.

We also calculated the formation energies of dimeric complexes extracted from the X-ray structures (Supplementary Table 2), which provide some further insight into the strength of the π-stacking interaction between the Pt-pincer complexes and the hydrogen bonding between the solvents and the ancillary ligands. The formation energy of the Form-II complex (i.e. a π-stacked Pt-pincer dimer) gives an estimate of the strength of the π-stacking interaction between pairs of pincer complexes. PBEsol predicts a low complexation energy of ~10 kJ mol^{-1} per molecule, whereas the dispersion-corrected PBEsol and PBE0 calculations yield much larger energies of ~56 and 72 kJ mol^{-1} per molecule, respectively. As with the solid-state formation energies, this suggests that the dispersion correction, and perhaps also the more accurate hybrid PBE0 functional, afford a better description of the interaction between pincer complexes.

Comparing the relative formation energies of the Form-I/Form-III and Form-II complexes affords an estimate of the strength of the hydrogen-bonding interaction between the cyanide ligand on the pincer complexes and water/methanol. The comparison yields values of ~25-40 kJ mol^{-1} per bond to methanol, and around 15-25 kJ mol^{-1} per bond to water. Although the strength of the bonds to water are calculated to be weaker, in the solid each molecule forms hydrogen bonds to two adjacent pincer complexes, whereas the methanol molecules in the Form-III solid interact with a single complex. Interestingly, whereas PBEsol and PBEsol + D3 (BJ) predict similar relative formation energies for the two hydrogen-bonded complexes, the PBE0 + D3(BJ) calculations predict energies ~15-20 kJ mol^{-1} higher. This may be ascribed to the in principle more accurate description of electrostatic interactions afforded by PBE0.

Supplementary Methods

Synthesis of methyl 3,5-di(2-pyridyl)benzoate

The polypyridine ligand 3,5-di(2-pyridyl)benzoate was prepared using a Stille coupling according to a literature procedure.[21] Methyl-3,5-dibromobenzoate (2 g, 6.8 mmols), 2-tri-n-butylstannylpyridine (tech. 80 %; 6 mL, 15 mmols), bis(triphenylphosphine)palladium dichloride (300 mg, 0.43 mmol) and LiCl (2.8 g, 65.4 mmol) were added to dry degassed toluene (30 mL) and the mixture was freeze-pump-thaw degassed for five cycles. The mixture was then heated under reflux at 120 °C for 24 hrs under an inert atmosphere. The reaction mixture was allowed to cool to room temperature before a saturated aqueous solution of KF was added to quench the reaction. The resulting slurry was filtered over diatomaceous earth, washed with toluene (10 mL) and the eluent retained and dried under vacuum. To the resulting residue, DCM (150 mL) was added, and the solution was purified twice with 100 mL of 5 % aqueous $NaHCO_3$ solution, with the DCM layer being retained each time. The solution was then dried with anhydrous $MgSO_4$, filtered, and the solvent removed under reduced pressure. The resulting mixture was then purified in a silica column with gradient elution of hexane to 1:4 hexane:Et_2O, resulting in fluffy white powder as product (42 %).

The alternative procedure based on a Negishi coupling was performed as follows. Methyl-3,5-dibromobenzoate (1.0 g, 3.4 mmol), 1,1'-ferrocenediyl-bis(diphenylphosphine) (dppf; 0.07 g, 0.12 mmol), bis(dibenzylideneacetone)palladium ($Pd(dba)_2$; 0.075 g, 0.135 mmol) were charged to a flame-dried flask to which was added dry THF (12 mL) and 0.5 M pyridylzinc bromide (27.2 mL, 6.08 mmol). The dark-brown solution was refluxed at 75 °C under nitrogen for 12 hrs, after which the solution was cooled and the THF removed under vacuum. The remaining solid was purified via flash chromatography using dry loading and a 1:3 petroleum ether:2 % triethylamine in ethyl acetate mixture as eluent (0.59 g, 60 %).

Characterisation of methyl 3,5-di(2-pyridyl)benzoate

^{1}H NMR (400 MHz, $CDCl_3$) δH (pm): 8.98 (t, $^4J_{H-H}$ = 1.8 Hz, 1H, para-Ph), 8.80 (d, $^3J_{H-H}$ = 4.8 Hz, 2H, ortho-Py), 8.77 (d, $^4J_{H-H}$ = 1.8 Hz, 2H, ortho-Ph), 7.97 (d, $^3J_{H-H}$ = 8.0 Hz, 2H, meta-Py), 7.86 (td, $^3J_{H-H}$ = 7.7 Hz, $^4J_{H-H}$ =1.8 Hz, 2H, para-Py), 7.36 (dd, $^3J_{H-H}$ = 4.8 Hz, $^4J_{H-H}$ = 1.1 Hz, 1H, meta-Py), 7.34 (dd, $^3J_{H-H}$ = 4.8 Hz, $^4J_{H-H}$ =1.1 Hz, 1H, meta-Py), 4.04 (s, 3H, $COOCH_3$).

Synthesis of chloro[methyl 3,5-di(2-pyridyl)benzoato]platinum

To a stirred solution of methyl-3,5-di(2-pyridyl)benzoate (100 mg, 0.34 mmol) in acetonitrile (15 mL) was added a solution of $K_2[PtCl_4]$ (144 mg, 0.34 mmol) in water (5 mL), and the resulting orange mixture was refluxed at 100 °C for 3 days. The reaction mixture was then allowed to cool, resulting in a vivid yellow precipitate which was retained in a filter and washed with water (20 mL), ethanol (20 mL), acetonitrile (20 mL), and Et_2O (10 mL). The product was collected as a bright yellow solid (98 mg, 55.4 % yield)

Characterisation of chloro[methyl 3,5-di(2-pyridyl)benzoato]platinum

^{1}H NMR (500 MHz, $CDCl_3$) δH (ppm): 9.36 (d, $^3J_{H\text{-}H}$ = 4.8 Hz, Pt satellite: $^3J_{H\text{-}Pt}$ = 41.1 Hz, 2H, ortho-Py), 8.12 (s, 2H, pincer-Ph), 8.01 (td, $^3J_{H\text{-}H}$ = 7.8 Hz, $^4J_{H\text{-}H}$ = 1.4 Hz, 2H, para-Py), 7.81 (d, $^3J_{H\text{-}H}$ = 7.9 Hz, 2H, meta-Py), 7.36 (td, $^3J_{H\text{-}H}$ = 5.7 Hz, $^4J_{H\text{-}H}$ = 1.4 Hz, 2H, meta-Py), 3.97 (s, 3H, CO_2CH_3).

*Synthesis of complex **1***

To a stirred suspension of freshly prepared AgCN (25.7 mg, 0.192 mmol) in 30ml of acetonitrile and dichloromethane (1:1 v/v) was added chloro[methyl 3,5-di(2-pyridyl)benzoato]platinum (100 mg, 0.192 mmol). The reaction mixture was heated to 85 °C under reflux for 24 hrs under a nitrogen atmosphere. The resulting orange/brown suspension was dried under reduced pressure, and then stirred with dichloromethane (30 mL) until the sparingly soluble residue was dissolved. The yellow solution was then filtered, and the solvent removed under vacuum. A bright red solid was obtained as crude product, washed with an excess of diethyl ether, and recrystallised by dissolving in boiling methanol, filtering, and cooling slowly (31 % yield).

Smart coatings on glass

Thin films of complex **1** on microscope glass slides (Fig. 3 (a)) were prepared by drop-casting a dichloromethane solution and allowing the solvent to evaporate. Similar films were prepared on highly-ordered pyrolytic graphic (HOPG) substrates by drop-casting a solution of **1** in chloroform. The microstructure of the HOPG films was then analysed by scanning-electron microscopy (SEM) using a JEOL SEM6480LV microscope operated at 10 kV (Fig. 3 (f), text).

Water vapour sensitivity measurements

To estimate the sensitivity to water vapour, we carried out a standard LiCl test as follows. A drop-cast film of **1** was prepared on a glass slide as outlined above and attached face-down to a sealed three-neck round-bottom flask containing a LiCl(aq) slurry. A thermocouple (Thor Labs TSP01) was inserted through a rubber seal to allow the temperature of the solution to be monitored. The flask was kept in dry ice, evacuated and filled with dry nitrogen to convert the film to the yellow Form-II, and then sealed at 1 atm. The vessel was transferred to a Polar Bear Plus (Cambridge Reactor Design), used to control the temperature of the slurry, and a light source and probe were positioned at 45 ° to one another to collect diffuse-reflectance spectra of the film through the supporting glass slide (Ocean Optics Maya 2000 Pro with DL-2000-BAL balanced deuterium-tungsten light source and QP600-2-SR-BX 600 μm premium fibres with solarisation-resistant jacket; 220-1100 nm spectral range, 100 ms exposure time, 10 scan accumulation, 10-point boxcar smoothing). Before taking measurements, the spectrometer was calibrated against a spectralon standard (Ocean Optics WS-1-SL diffuse-reflectance standard). The setup is shown in the photograph in Supplementary Figure 4.

The temperature of the solution was raised incrementally from 4 to 40 °C, being monitored using the thermocouple, while reflectance spectra were recorded continuously. The film was observed to change from the yellow anhydrous Form-II to the red hydrated Form-I over the range of approx. 29-34 °C, which were converted to water vapour concentrations of ~4,500-6,000 ppmV (Supplementary Figures 5 and 6) using the data in Ref. [22]. The reflectance spectra were post-processed using a custom code, written in the Python programming language[23] with the NumPy,[24] SciPy[25] and Matplotlib[26] packages, and were smoothed with 21-point triangle filter and differentiated once using spline interpolation. This code has been made open-source and can be obtained from a public GitHub repository.[27]

Under controlled increase of the water vapour concentration, the spectroscopy also provides additional insight into the switching mechanism.

The first-derivative diffuse-reflectance spectra over a range of temperatures (equivalently, water vapour concentrations; Supplementary Figure 7) shows multiple features corresponding to the colour-transition edge of the yellow Form-II, *viz.* two peaks at ~530 and 560 nm, and a shoulder around 500 nm, along with a single peak corresponding to the transition edge of the red Form-I (~570-610 nm). As the water vapour concentration is increased over the range where the transition takes place, the features from Form-II diminish in intensity, while the maximum of the peak from Form-I grows and the maximum shifts continuously.

The continuous shift of the Form-I transition edge shows that, at intermediate water vapour concentrations, the crystallites can be “partially filled”, i.e. can contain less than one water molecule

per Pt-pincer complex. If the solvent molecules can diffuse through open channels in the crystal structure, this would allow the average Pt-Pt distance and/or the offset between adjacent complexes to vary continuously, thereby allowing for a continuous change in the absorption profile, as observed. This lends further support to the diffusive mechanism inferred from the structural analysis in the text.

The persistence of the features from Form-II suggests that, at intermediate water vapour concentrations, the film consists of a mixture of Form-II and Form-I crystallites (layering can be ruled out both from the films being relatively thin, and also based on the intensities of the spectral features[28]). This suggests that formation of the solvent channels requires a certain critical water vapour concentration, presumably dependent on the crystallite size, which is consistent with the conversion from Form-II to Form-I beginning with the solvent adsorbing to the crystal surface and then facilitating the formation of an initial channel structure for subsequent rapid diffusion.

Cycling endurance tests

To test the cycling endurance, a coated glass slide was mounted on a motorised holder spinning at 0.6 Hz and passed through a dry nitrogen gas stream once per cycle to induce the Form-I ↔ Form-II transition (Supplementary Figure 8; see also the accompanying videos). The test was left running for approx. 6 hrs, during which the sample was switched close to 13,000 times. We observed no visual difference between the vapochromic responses at the start and end of the test, indicating that the coated films can endure a large number of switching cycles while retaining their vapochromic functionality.

Impregnated polymer membranes

Supported membranes of the polymer PIM-EA-TB impregnated with complex **1** were grown on white polyethylene terephthalate (PET) films of 0.25 mm thickness (Goodfellow Cambridge, Ltd). PIM-EA-TB was first prepared following a literature procedure.[29] A 2-3% w/v solution of PIM-EA-TB and complex **1** was then prepared by dissolving 12 mg of a 10:1 mixture of PIM-EA-TB:**1** in 0.5 mL of $CHCl_3$. The solution was then poured into a 20 mm circular Teflon mould clamped to the PET substrate, and the solvent evaporated over 24 hrs at room temperature under a chloroform atmosphere. After preparation, the film could be removed from the support, yielding a free-standing membrane containing impregnated microcrystallites of **1** (Figs. 3 (g, h)). The microstructure of the supported membranes was studied using optical microscopy (GXML 3230 equipped with a GXCAM USB HiChrome-S digital camera and the GXCapture software; Supplementary Figure 9).

Growth of single crystals for X-ray diffraction studies

Red crystals with a needle morphology, consistent in appearance with the bulk powder of Form-I, were grown by slow evaporation of an acetonitrile solution under ambient conditions (i.e. in the presence of atmospheric water vapour). The structure was characterised using single-crystal X-ray diffraction, and a simulated powder pattern matched that recorded from the bulk solid powder, confirming it to be a representative structure (Supplementary Figure 10).

Blue crystals with a needle morphology, consistent in appearance with Form-III, were obtained from a slow-cooled methanol solution, and were similarly confirmed to be structurally representative of the bulk solid by matching simulated and recorded powder patterns (Supplementary Figure 11). These crystals could not be kept in ambient air due to conversion to the hydrated Form-I. To collect an X-ray structure, a methanol suspension of the needle crystals was injected into a layer of Fomblin oil, and a single crystal was mounted suspended in a droplet of oil onto a micromount. This was then flash cooled using the cryostream mounted on the diffractometer, effectively avoiding exposure to ambient air during data collection.

Four different yellow needle-like crystals consistent in appearance with Form-II were obtained by various crystallisation methods. Crystallising from a slow-cooled ethanol solution and by slow evaporation of an acetonitrile solution under inert atmosphere both yielded solvates containing one molecule of the respective solvent per formula unit and produced powder patterns structurally inconsistent with those of bulk powders of Form-II. Solvent layering of diethyl ether onto a dry dichloromethane solution produced yellow solvent-free crystals, but these again yielded a powder pattern inconsistent with the bulk material. None of these three crystals displayed vapochromic behaviour.

Slow evaporation of a dilute acetone solution under dry nitrogen yielded a fourth set of yellow crystals. These were found to be solvent free, with a powder pattern consistent with the bulk solid (Supplementary Figure 12). As with the Form-III crystals, careful preparation was required in order to avoid exposure to ambient air. An acetone suspension was injected into a layer of Fomblin oil, as with Form-III, and the sample was frozen on the mount in-situ on the microscope stage using liquid nitrogen before moving it to the diffractometer.

Single-crystal X-ray crystallographic studies

Crystallographic data for the X-ray structures of Form-I, Form-II and Form-III is summarised in Supplementary Table 1, the atomic arrangements in the asymmetric units are shown in Supplementary Figures 10-12, and the structures are available in Crystallographic Information File

(CIF) format from the Cambridge Crystal Structure Database (CCSD) under the refcodes 954459, 954461 and 1493352.

The structures of Form-I, Form-II and Form-III were collected at station 11.3.1, Advanced Light Source, Lawrence Berkeley National Laboratory, CA, USA, using a Bruker APEX II CCD diffractometer equipped with a 700 Series Oxford Cryosystems cryostream. Powder diffraction experiments for fingerprinting and the structures of the other crystals (inconsistent with the bulk powder) were recorded using an Oxford Diffraction Gemini A Ultra diffractometer equipped with an Oxford Cryosystems Cryojet XL 2.

Identification of natural crystal faces in Form I by face indexation

A needle crystal of the red Form I was selected and mounted on the diffractometer, as shown in Supplementary Figure 20 (a). Particular care was taken to ensure that the natural faces of the crystal were not damaged by manipulations, and the crystal was not cut to size in any dimension. Face indexation was then performed using the "Abs Display" tool in the Rigaku-Oxford Diffraction software CrysAlis Pro[10] (Supplementary Figure 20 (b)).

X-ray Single Crystal Structure Determination of the ethanol solvate form of complex **1**.EtOH

Crystals of the ethanol solvate of complex **1** (**1**.EtOH) were obtained by slow evaporation from a saturated solution of **1** in ethanol. The single-crystal X-ray structure confirms that one equivalent of ethanol per Pt(II)-pincer molecule is included in the crystal (see Supplementary Figure 21). **1**.EtOH crystallises in the same $P2_1/n$ space group as Forms I to III, but with a significantly larger unit cell volume due to the inclusion of the bulkier ethanol solvent molecule. The unit cell expansion is largely isotropic, and the structure of **1**.EtOH can be considered to be an expanded analogue of the other solvated forms of **1** reported in this study. As for the methanolic Form-III, the ethanol molecule is involved in a single, discrete hydrogen bonding interaction to N(1) of the CN- ligand. However, unlike Form-III, the more sterically-demanding ethanol molecule cannot be included in the crystal structure without disrupting the Pt…Pt overlap between adjacent pincer molecules in the stack. Supplementary Figure 22 shows that there is now no significant d_{z^2} overlap within the stack, which explains the yellow colour of the **1**.EtOH crystals.

Supplementary References

[1] CrysAlis Pro data collection and data reduction GUI version 171.38.43 (2017). Rigaku-Oxford Diffraction, https://www.rigaku-oxford.com/ (accessed 2017-06-02)

[2] Kresse, G. and Hafner, J. ABINITIO MOLECULAR-DYNAMICS FOR LIQUID-METALS. *Physical Review B* **47**, 558(R) (**1993**)

[3] Perdew, J. P., Ruzsinszky, A., Csonka, G. I., Vydrov, O. A., Scuseria, G. E., Constantin, L. A., Zhou, X. and Burke, K. Restoring the Density-Gradient Expansion for Exchange in Solids and Surfaces. *Physical Review Letters* **100**, 136046 (**2008**)

[4] Grimme, S., Antony, J., Ehrlich, S. and Krieg, H. A consistent and accurate *ab initio* parameterisation of density functional dispersion correction (DFT-D) for the 94 elements H-Pu, *The Journal of Chemical Physics* **132**, 154104 (**2010**)

[5] Grimme, S., Ehrlich, S. and Goerigk, L. Effect of the damping function in dispersion corrected density functional theory. *Journal of Computational Chemistry* **32**, 1456 (**2011**)

[6] Adamo, C. and Barone, V. Toward reliable density functional methods without adjustable parameters: The PBE0 model. *The Journal of Chemical Physics* **110**, 6158 (**1999**)

[7] Frisch, M. J., Trucks, G. W., Schlegel, H. B., Scuseria, G. E., Robb, M. A., Cheeseman, J. R., Scalmani, G., Barone, V., Mennucci, B., Petersson, G. A., Nakatsuji, H., Caricato, M., Li, X., Hratchian, H. P., Izmaylov, A. F., Bloino, J., Zheng, G., Sonnenberg, J. L., Hada, M., Ehara, M., Toyota, K., Fukuda, R., Hasegawa, J., Ishida, M., Nakajima, T., Honda, Y., Kitao, O., Nakai, H., Vreven, T., Montgomery, J. A. Jr., Peralta, J. E., Ogliaro, F., Bearpark, M. J., Heyd, J., Brothers, E. N., Kudin, K. N., Staroverov, V. N., Kobayashi, R., Normand, J., Raghavachari, K., Rendell, A. P., Burant, J. C., Iyengar, S. S., Tomasi, J., Cossi, M., Rega, N., Millam, N. J., Klene, M., Knox, J. E., Cross, J. B., Bakken, V., Adamo, C., Jaramillo, J., Gomperts, R., Stratmann, R. E., Yazyev, O., Austin, A. J., Cammi, R., Pomelli, C., Ochterski, J. W., Martin, R. L., Morokuma, K., Zakrzewski, V. G., Voth, G. A., Salvador, P., Dannenberg, J. J., Dapprich, S., Daniels, A. D., Farkas, Ö., Foresman, J. B., Ortiz, J. V., Cioslowski, J. and Fox, D. J. Gaussian 09. (Wallingford, CT, USA: Gaussian, Inc., 2009)

[8] Hohenberg, P. and Kohn, W. INHOMOGENEOUS ELECTRON GAS. *Physical Review B* **136**, 864 (**1964**)

[9] Kohn, W. and Sham, L. J. SELF-CONSISTENT EQUATIONS INCLUDING EXCHANGE AND CORRELATION EFFECTS. *Physical Review* **140**, 1133 (**1965**)

[10] Hanwell, M. D., Curtis, D. E., Lonie, D. C., Vandermeersch, T., Zurek, E. and Hutchison, G. R. Avogadro: an advanced semantic chemical editor, visualization, and analysis platform. *Journal of Cheminformatics* **4** (**2012**)

[11] Ditchfield, R., Hehre, W. J. and Pople, J. A. Self-Consistent Molecular-Orbital Methods. IX. An Extended Gaussian-Type Basis for Molecular-Orbital Studies of Organic Molecules. *The Journal of Chemical Physics* **54**, 724 (**1971**)

[12] Hay, P. J. and Wadt, W. R. *Ab initio* effective core potentials for molecular calculations. Potentials for K to Au including the outermost core orbitals. *The Journal of Chemical Physics* **82**, 299 (**1985**)

[13] Hay, P. J. and Wadt, W. R. *Ab initio* effective core potentials for molecular calculations. Potentials for the transition metal atoms Sc to Hg. *The Journal of Chemical Physics* **82**, 270 (**1985**)

[14] Reed, A. E., Weinstock, R. B. and Weinhold, F. Natural-Population Analysis. *The Journal of Chemical Physics* **83**, 735 (**1985**)

[15] Giannozzi, P., Baroni, S., Bonini, N., Calandra, M., Car, R., Cavazzoni, C., Ceresoli, D., Chiarotti, G. L., Cococcioni, M., Dabo, I., Dal Corso, A., de Gironcoli, S., Fabris, S., Fratesi, G., Gebauer, R., Gerstmann, U., Gougoussis, C., Kokalj, A., Lazzeri, M., Martin-Samos, L., Marzari, N., Mauri, F., Mazzarello, R., Paolini, S., Pasquarello, A., Paulatto, L., Sbraccia, C., Scandolo, S., Sclauzero, G., Seitsonen, A. P., Smogunov, A., Umari, P. and Wentzcovitch, R. M. QUANTUM ESPRESSO: a modular and open-source software project for quantum simulations of materials. *Journal of Physics: Condensed Matter* **21**, 395502 (**2009**)

[16] Blochl, P. E. PROJECTOR AUGMENTED-WAVE METHOD. *Physical Review B* **50**, 17953 (**1994**)

[17] Kresse, G. and Joubert, D. From ultrasoft pseudopotentials to the projector augmented-wave method. *Physical Review B* **59**, 1758 (**1999**)

[18] Monkhorst, H. J. and Pack, J. D. SPECIAL POINTS FOR BRILLOUIN-ZONE INTEGRATIONS. *Physical Review B* **13**, 5188 (**1976**)

[19] Gajdos, M., Hummer, K., Kresse, G., Furthmuller, J. and Bechstedt, F. Linear optical properties in the projector-augmented wave methodology. *Physical Review B* **73**, 045112 (**2006**)

[20] Skelton, J. M., da Silva, E. L., Crespo-Otero, R., Hatcher, L. E., Raithby, P. R., Parker, S. C. and Walsh, A. Electronic excitations in molecular solids: bridging theory and experiment. *Faraday Discussions* **177**, 181 (**2015**)

[21] Williams, J. A. G., Beeby, A., Davies, E. S., Weinstein, J. A. and Wilson, C. An Alternative Route to Highly Luminescent Platinum(II) Complexes: Cyclometalation with NCN-Coordinating Dipyridylbenzene Ligands. *Inorganic Chemistry* **42**, 8609 (**2003**)

[22] https://www.omega.co.uk/temperature/Z/pdf/z103.pdf and http://www.humcal.com/index.php (accessed 2017-03-31)

[23] Python Software Foundation, https://www.python.org/ (accessed 2017-06-02)

[24] van der Walt, S., Colbert, S. C and Varoquaux, G. The NumPy Array: A Structure for Efficient Numerical Computation. *Computing in Science & Engineering* **13**, 22 (**2011**)

[25] Oliphant, T. E. Python for Scientific Computing. *Computing in Science & Engineering* **9**, 10 (**2007**)

[26] Hunter, J. D. Matplotlib: A 2D Graphics Environment. *Computing in Science & Engineering* **9**, 90 (**2007**)

[27] MayaPy, https://github.com/JMSkelton/MayaPy (accessed 2017-06-02)

[28] Pallipurath, A. R., Skelton, J. M., Ricciardi, P., Elliott, S. R. Estimation of semiconductor-like pigment concentrations in paint mixtures and their differentiation from paint layers using first-derivative reflectance spectra. *Talanta* **154**, 63 (**2016**)

[29] Carta, M., Malpass-Evans, R., Croad, M., Rogan, Y., Jansen, J. C., Bernardo, P., Bazzarelli, F. and McKeown, N. B. An Efficient Polymer Molecular Sieve for Membrane Gas Separations. *Science* **339**, 303 (**2013**)
