# Supplementary material for: A rapidly-reversible absorptive and emissive vapochromic Pt(II) pincer-based chemical sensor
Source: Nat Commun. 2017 Nov 27;8:1800. doi: 10.1038/s41467-017-01941-2 (PMC5702612; doi:10.1038/s41467-017-01941-2)
Supplement: Supplementary file 3 — Description of Additional Supplementary Files [file 41467_2017_1941_MOESM3_ESM.pdf]

Description of Additional Supplementary Files

File Name: Supplementary Movie 1
Description: Switching of a film of 1 coated onto glass between Form-I (hydrated, red) to Form-II (anhydrous, yellow) under a dry nitrogen stream.

File Name: Supplementary Movie 2
Description: Switching of a film on glass between Form-I and Form-III (methanolic, blue) by exposure to liquid methanol.

File Name: Supplementary Movie 3
Description: Switching of a film on glass between Form-III and Form-II under a dry nitrogen stream. The film reverts to Form-I once the stream is removed after purging the methanol.

File Name: Supplementary Movie 4
Description: Switching of a film on glass between Form-I to Form-II under UV, showing the resulting change in emissive colour.

File Name: Supplementary Movie 5
Description: Video of the setup used to test the switching durability of the films on glass.
