# Supplementary material for: A rapidly-reversible absorptive and emissive vapochromic Pt(II) pincer-based chemical sensor
Source: Nat Commun. 2017 Nov 27;8:1800. doi: 10.1038/s41467-017-01941-2 (PMC5702612; doi:10.1038/s41467-017-01941-2)
Supplement: Supplementary file 2 — Peer Review File [file 41467_2017_1941_MOESM2_ESM.pdf]

**Editorial Note:** This manuscript has been previously reviewed at another journal that is not operating a transparent peer review scheme. The manuscript was considered suitable for publication without further review at *Nature Communications.*
